# Distinct nucleus accumbens neural pathways underlie separate behavioral features of chronic pain and comorbid depression

**DOI:** 10.1172/JCI191270

**Published:** 2025-08-01

**Authors:** Di Liu, Fang-Xia Xu, Zhuang Yu, Xiao-Jing Huang, Ya-Bing Zhu, Li-Juan Wang, Chen-Wei Wu, Xu Zhang, Jun-Li Cao, Jinbao Li

**Affiliations:** 1 Department of Anesthesiology, Shanghai General Hospital, Shanghai Jiao Tong University School of Medicine, Shanghai, China.; 2 Department of Pain Medicine, Shanghai Geriatric Medical Center, Shanghai, China.; 3 School of Anesthesiology, Shandong Second Medical University, Weifang, China.; 4 Jiangsu Province Key Laboratory of Anesthesiology and Jiangsu Province Key Laboratory of Anesthesia and Analgesia Application Technology, National Medical Products Administration Key Laboratory for Research and Evaluation of Narcotic and Psychotropic Drugs, Xuzhou Medical University, Xuzhou, China.; 5 Department of Anesthesiology, The Affiliated Hospital of Xuzhou Medical University, Xuzhou, China.

**Keywords:** Neuroscience, Therapeutics, Behavior, Pain, Psychiatric diseases

## Abstract

The comorbidity of depressive symptoms in chronic pain has been recognized as a key health issue. However, whether discrete circuits underlie behavioral subsets of chronic pain and comorbid depression has not been addressed. Here, we report that dopamine 2 (D2) receptor–expressing medium spiny neurons in the nucleus accumbens medial shell (mNAcSh) mediate pain hypersensitivity and depression-like behaviors in mice after nerve injury. Two separate neural pathways mediate different symptoms. The glutamatergic inputs from the anteromedial thalamic nucleus to mNAcSh D2 neurons that innervated orexin-expressing neurons in the lateral hypothalamic area contributed to pain regulation. In contrast, the lateral septum GABAergic inputs to mNAcSh D2 neurons that disinhibit the ventral pallidum glutamatergic neurons mediated depression-like behaviors. These findings indicate the functional significance of heterogeneous mNAcSh D2 neurons and their neural pathways, providing a perspective for symptom-specific treatments of chronic pain and comorbid depression.

## Abstract 2

Divergent nucleus accumbens neural circuits differentially contributed to chronic pain and comorbid depressive-like bahviors.

## Graphical abstract

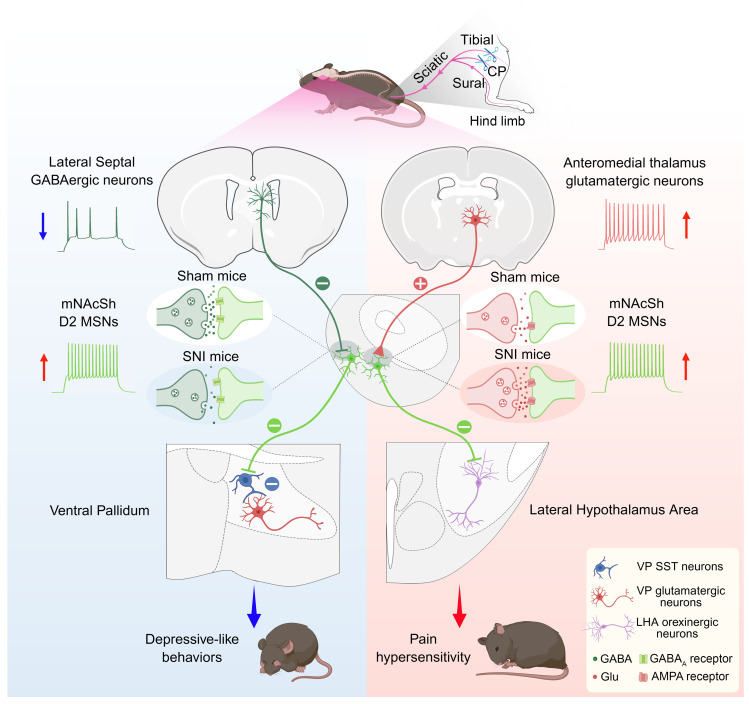

## Introduction

Pain comorbid with depression is frequently encountered in patients and often coexacerbates physical and psychological symptoms (1, 2). Conversely, negative emotions further induce more pain complaints and worsen pain perception (3, 4). Multiple lines of evidence demonstrate the shared neural circuits underlying pain and comorbid emotional disorders (5–7). However, controversial results have been reported in studies concerning the analgesic effect of antidepressants (8–10). Such differences may be explained by the existence of discrete neural circuits underlying chronic pain and comorbid depression. Indeed, recent studies have demonstrated specific neural projections underlying psychiatric disorders associated with chronic pain and unmasked distinct neural pathways mediating separate symptoms of depression (11, 12). However, it remains unknown how different circuits are interrelated and consequently contribute to discrete behavioral domains of chronic pain and comorbid depression.

The ventral striatum nucleus accumbens (NAc) is an important convergent point at the interface of cognitive and emotional circuits, and its dysfunction has been implicated in both pain and depressive disorders (13–15). Studies have identified structural and functional alterations in the NAc under chronic pain conditions (16–19), indicating a potential neural basis for the NAc to contribute to pain management (20–22). Additionally, metabolic and neurotrophic signaling mechanisms in the NAc have been demonstrated to regulate depressive behaviors in animal models (23–25). The NAc receives inputs from the ventral tegmental area, paraventricular thalamus (PVT), amygdala, and prefrontal cortex — critical central regions for pain or depression modulation (13, 26–29). Given its connections to these regions, the NAc is a likely target capable of integrating and transmitting pain- and depression-relevant signals across the brain. However, it is still unknown how the NAc circuitry contributes to the comorbidity of chronic pain and depression. Moreover, the NAc exhibits considerable cellular, anatomic, and functional heterogeneity in regulating multiple behaviors (13, 30), and the modulatory effects of NAc neurons on pain sensation are contradictory in different studies (19, 22, 31). Thus, it is critical to dissect the architecture and function of the distinct subpopulations of NAc that contribute to diverse features of chronic pain and depression-like behaviors.

Our results in this study reveal that the medial shell region of the NAc (mNAcSh) is involved in mediating multifaceted depression-like behaviors in mouse models of chronic pain. This can be attributed to the activation of dopamine 2–expressing (D2-expressing) mNAcSh medium spiny neurons (MSNs). Inhibition of the mNAcSh D2 neurons substantially alleviates chronic pain and depression-like behaviors. Moreover, 2 separate subpopulations of D2 neurons, through their respective projections to the lateral hypothalamic area (LHA) and ventral pallidum (VP), mediate chronic pain and depressive disorders. These separate mNAcSh D2 GABAergic outputs lead to inhibition of LHA orexinergic neurons and disinhibition of VP glutamatergic neurons. Finally, the LHA-projecting mNAcSh D2 neurons (mNAcSh^D2^-LHA) are preferably innervated by glutamatergic neurons of the anteromedial thalamic nucleus (AM) and mediate pain hypersensitivity. In contrast, the VP-projecting mNAcSh D2 neurons (mNAcSh^D2^-VP) are selectively innervated by the lateral septum (LS) GABAergic neurons and mediate depression-like phenotypes. Together, these results elucidate the contribution of discrete mNAcSh D2 neural circuits to specific behavioral subdomains of chronic pain and comorbid depression-like states.

## Results

### The mNAcSh D2 neurons are activated in neuropathic pain mice comorbid with depression-like phenotypes.

**Figure 1:**
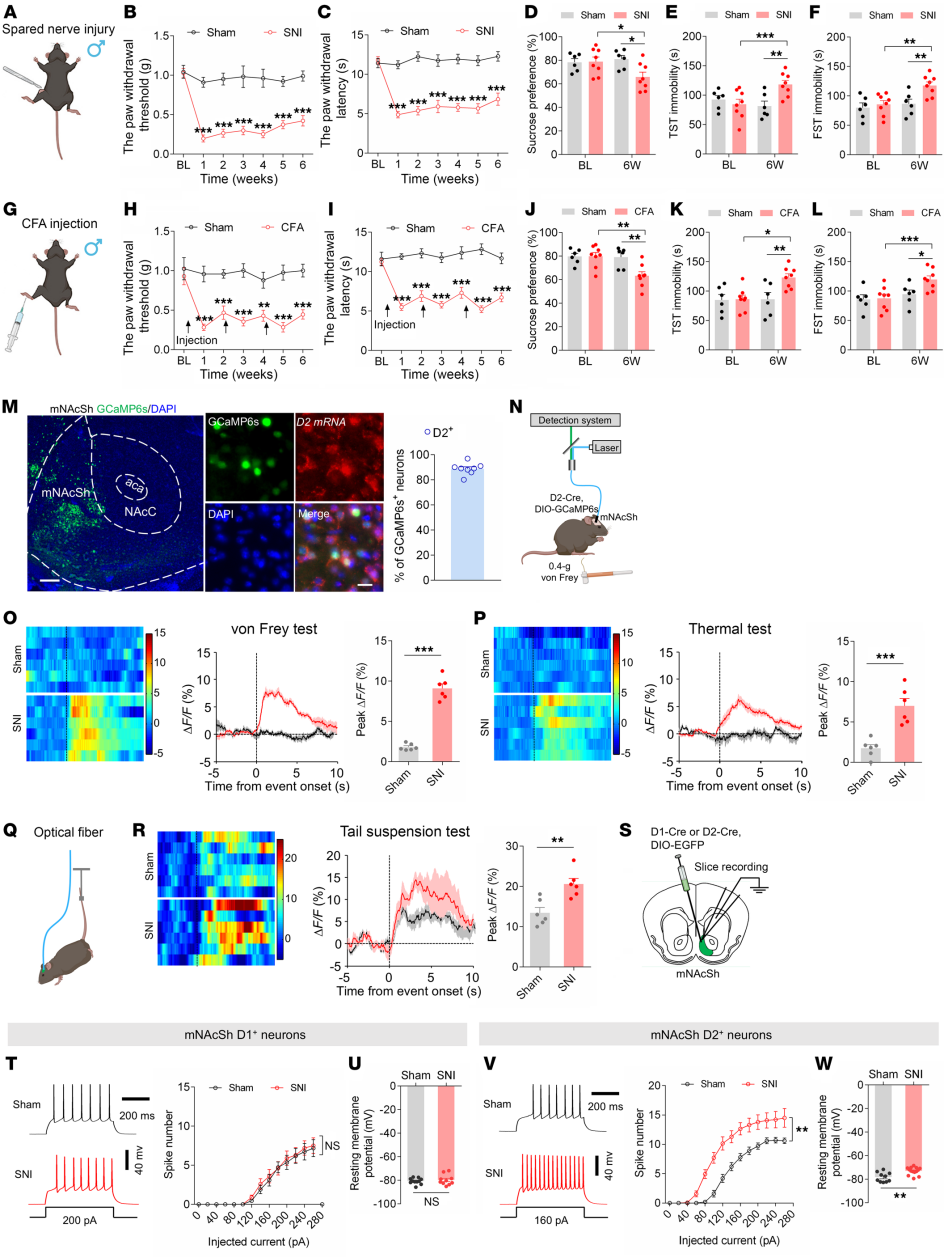
Activation of mNAcSh D2 neurons in neuropathic pain mice comorbid with depression. (**A**) Schematic of spared nerve injury. (**B** and **C**) Mechanical (**B**) and thermal (**C**) pain thresholds in sham and SNI mice (*n* = 6–8 mice/group); interaction *F*_(6, 72)_ = 6.873 (**B**); *F*_(6, 72)_ = 9.344 (**C**). BL, baseline. (**D**–**F**) Sucrose preference (**D**), immobility time in TST (**E**), and FST (**F**) in mice (*n* = 6–8 mice/group); *F*_(1, 12)_ = 5.435 (**D**); *F*_(1, 12)_ = 21.21 (**E**); *F*_(1, 12)_ = 5.355 (**F**). (**G**) Injection schematic. (**H** and **I**) Mechanical (**H**) and thermal (**I**) pain thresholds (*n* = 6–8 mice/group); *F*_(6, 72)_ = 3.226 (**H**); *F*_(6, 72)_ = 8.248 (**I**). (**J**–**L**) Sucrose preference (**J**), immobility time in TST (**K**), and FST (**L**) in mice (*n* = 6–8 mice/group); *F*_(1, 12)_ = 8.868 (**J**); *F*_(1, 12)_ = 9.891 (**K**); *F*_(1, 12)_ = 5.157 (**L**). (**M**) D2 mRNA expression in GCaMP6s-positive neurons; *n* = 8. Scale bars: 200 μm (left), 20 μm (right). aca, anterior commissure, anterior part. (**N** and **Q**) Schematic showing photometry. (**O**, **P**, and **R**) Heat maps, average, and quantification of Δ*F*/*F* illustrating Ca^2+^ signals upon von Frey (**O**), thermal stimuli (**P**), and tail suspension (**R**) (*n* = 6 mice/group). (**S**) Schematic showing electrophysiological recording. (**T** and **V**) The spikes in response to currents injection in D1 (**T**) and D2 (**V**) neurons (**T**, *n* = 10–11 neurons/group, *F*_(13, 247)_ = 0.1533; and **V**, *n* = 11–12 neurons/group, *F*_(1,22)_ = 12.98). (**U** and **W**) RMP of D1 (**U**) and D2 (**W**) neurons (**U**, *n* = 10–11; **W**, *n* = 11–12). *P < 0.05, **P < 0.01, ***P < 0.001 by 2-way repeated measures ANOVA with Bonferroni’s multiple comparisons test (**B**–**F**) and (**H**–**L**) or 2-tailed *t* test (**O**, **P**, **R**, **U**, and **W**). Data are represented as mean ± SEM.

To explore the effects of persistent pain on depression-like behaviors in mice, we first exploited a well-established rodent model of neuropathic pain using spared nerve injury (SNI) surgery in male mice (32) (Figure 1A). SNI mice exhibited persistent (up to 6 weeks) decreases in paw withdrawal threshold and paw withdrawal latency compared with sham mice (Figure 1, B and C). Specifically, at 6 weeks after SNI surgery, the mice exhibited reduced sucrose preference in the sucrose preference test (that is, anhedonia) and increased immobility in the tail suspension test (TST) and forced swimming test (FST) (that is, helplessness) (Figure 1, D–F), indicating depression-like phenotypes (33). Our findings were consistent with previous studies (34, 35); thus, 6 weeks after SNI surgery was selected as the time point for assessment in this study. These phenotypes were also observed in an inflammatory pain model using the injection of CFA in mice (Figure 1, G–L). Since depression-like symptoms are notably related to neuropathic pain, which is a clinical challenge that needs to be urgently addressed, we mainly focused on the SNI mouse model in this study.

To examine the role of NAc in depression-like behaviors resulting from chronic pain, we first examined the expression of c-fos, a marker of neural activity, within different subregions of the NAc. In particular, 6 weeks after SNI surgery, the c-fos expression level in the mNAcSh, rather than NAc lateral shell (NAcLat) or NAc core (NAcCore), was dramatically higher than that after sham surgery (Supplemental Figure 1, A and B; supplemental material available online with this article; https://doi.org/10.1172/JCI191270DS1). Consistently, whole-cell recording in brain slices showed increased excitability in mNAcSh neurons of SNI 6-week mice compared with sham mice (Supplemental Figure 1, C–K). These results suggest that the mNAcSh neurons were activated in mice with chronic neuropathic pain.

The NAc is mostly composed of MSNs expressing dopamine 1 (D1) receptors and those expressing D2 receptors (36). Thus, we investigated how these 2 subpopulations respond to neuropathic pain and comorbid depression-like phenotypes. We first targeted the slow variant of genetically encoded calcium indicator (GCaMP6s) to mNAcSh D2 neurons by injecting a Cre-dependent recombinant adeno-associated virus (AAV) encoding GCaMP6s into the mNAcSh and implanted optical fibers in D2-Cre mice (Figure 1M). Morphological analysis revealed that GCaMP6s-expressing mNAcSh neurons were largely positive for D2 receptor mRNA (Figure 1M). To characterize the Ca^2+^ activity of mNAcSh D2 neurons associated with pain hypersensitivity and depression-like behaviors, we applied innocuous mechanical stimuli (0.4 g von Frey fiber), which scarcely evoked paw withdrawal responses in sham-operated mice yet caused marked withdrawal in mice with neuropathic pain (37). We observed enhanced activation of mNAcSh D2 neurons upon von Frey stimulation in post-SNI 6W mice (Figure 1, N and O). Consistently, their Ca^2+^ activity was increased in SNI mice in response to thermal stimulation (Figure 1P). Furthermore, the mNAcSh D2 neural Ca^2+^ activity was enhanced in the immobility period during TST in SNI mice when compared with sham-operated mice (Figure 1, Q and R). Interestingly, in vivo fiber photometry revealed an insignificant difference in the Ca^2+^ activity of mNAcSh D1 neurons during either pain-related stimuli or the TST (Supplemental Figure 2). We also conducted whole-cell brain slice recordings to test the membrane excitability of NAc neurons in mice (Figure 1S). The results revealed increased membrane excitability of the mNAcSh D2, but not D1, neurons of SNI 6-week mice compared with sham-operated mice (Figure 1, T–W). These findings align with previous electrophysiological studies demonstrating that the membrane excitability of NAc D2 neurons is increased in mice with neuropathic pain (19). Our findings suggested enhanced excitability of mNAcSh D2 neurons in mice with chronic neuropathic pain and comorbid depression.

### mNAcSh D2 neurons mediate neuropathic pain and depression-like behaviors.

**Figure 2:**
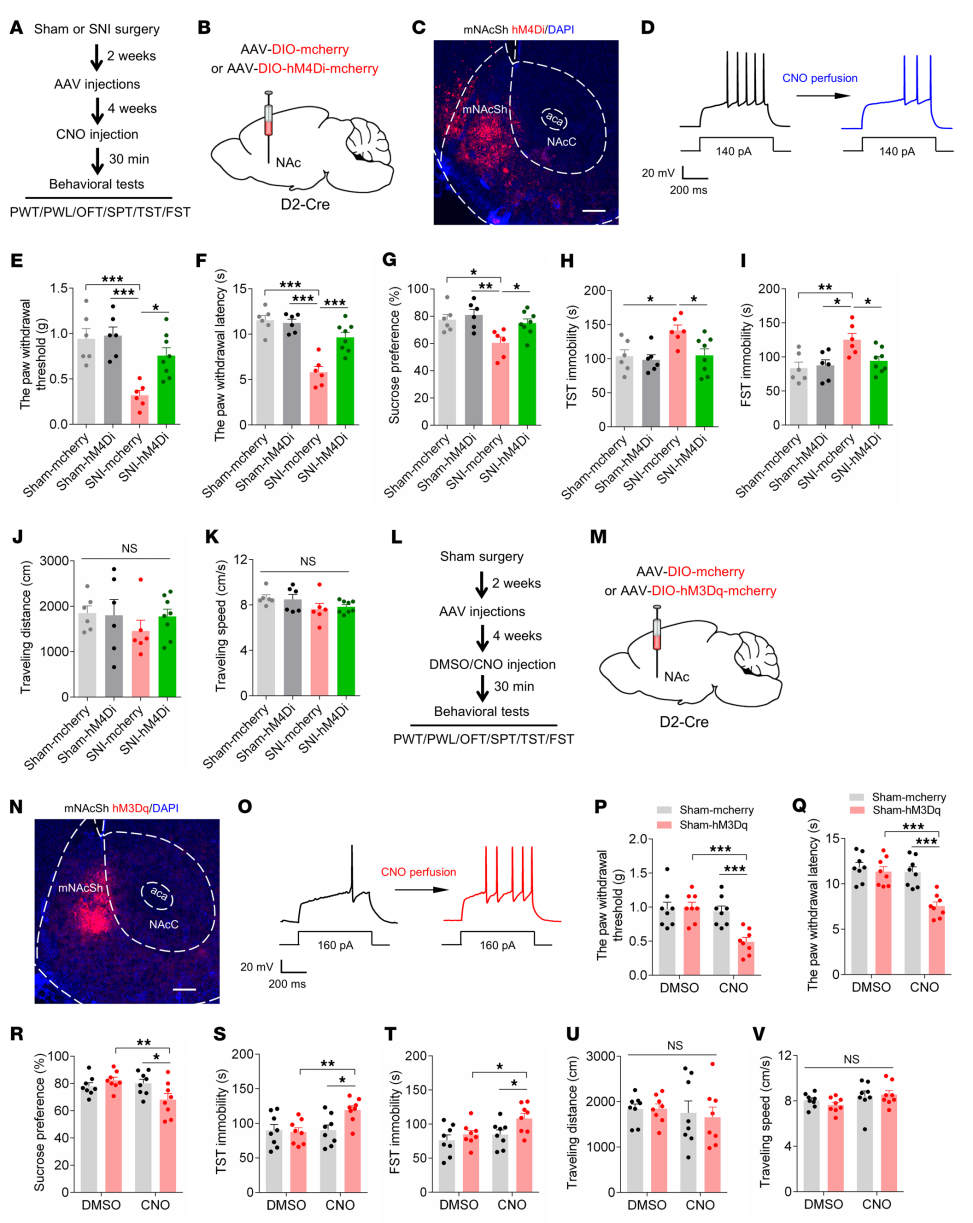
Manipulations of mNAcSh D2 neurons bilaterally modulates pain and depression-like behaviors in mice. (**A** and **B**) Time course (**A**) and schematic (**B**) showing virus injection and behavioral tests. (**C**) The example image showing hM4Di-mCherry expression in mNAcSh. Scale bar: 200 μm. (**D**) Slice recording of an hM4Di-expressing mNAcSh D2 neuron with CNO application. (**E** and **F**) Mechanical (**E**) and thermal (**F**) pain thresholds in mice that received i.p. injection of CNO (*n* = 6–8 mice/group); *F*_(1, 22)_ = 4.937 (**E**); *F*_(1, 22)_ = 13.92 (**F**). (**G**–**I**) Sucrose preference (**G**) and immobility time in TST (**H**) and FST (**I**) (*n* = 6–8 mice/group); *F*_(1, 22)_ = 9.377 (**G**); *F*_(1, 22)_ = 6.049 (**H**); *F*_(1, 22)_ = 4.563 (**I**). (**J** and **K**) The traveling distance (**J**) and average speed (**K**) in the open field test (OFT) (*n* = 6–8 mice/group); *F*_(1, 22)_ = 0.6301 (**J**); *F*_(1, 22)_ = 0.2234 (**K**). (**L** and **M**) The experimental design (**L**) and schematic of virus injection (**M**). (**N**) A representative image showing hM3Dq-mCherry expression. Scale bar: 200 μm. (**O**) Slice recording of an hM3Dq-expressing mNAcSh D2 neuron with CNO application. (**P** and **Q**) The mechanical (**P**) and thermal (**Q**) pain thresholds in sham-operated mice with chemogenetic activation of mNAcSh D2 neurons (*n* = 8 mice/group); interaction *F*_(1, 14)_ = 19.69 (**P**); *F*_(1, 14)_ = 13.87 (**Q**). **(R**–**V**) Sucrose preference (**R**), immobility time in TST (**S**) and FST (**T**), and traveling distance (**U**) and speed in the OFT (**V**) (*n* = 8 mice/group); interaction *F*_(1, 14)_ = 9.370 (**R**); *F*_(1, 14)_ = 7.181 (**S**); *F*_(1, 14)_ = 7.974 (**T**); *F*_(1, 14)_ = 0.05896 (**U**); *F*_(1, 14)_ = 1.095 (**V**). *P < 0.05, **P < 0.01, and ***P < 0.001 by 2-way ANOVA with Tukey’s multiple-comparison test (**E**–**K** and **P**–**V**). Data are represented as mean ± SEM.

Since increased Ca^2+^ response and enhanced neural excitability were demonstrated in mNAcSh D2 neurons, we further investigated the effects of manipulating these neurons on pain- and depression-related behaviors in SNI mice. We selectively expressed AAV encoding Cre-dependent inhibitory designer receptors exclusively activated by designer drugs (DREADD) hM4Di (or mCherry as control) in mNAcSh D2 neurons (Figure 2, A and B). Morphological and electrophysiological studies confirmed the functional hM4Di expression in the mNAcSh D2 neurons (Figure 2, C and D). The i.p. administration of the ligand clozapine-*N*-oxide (CNO) increased mechanical paw withdrawal threshold and thermal paw withdrawal latency in hM4Di-expressing SNI mice compared with the mCherry-expressing SNI animals (Figure 2, E and F). In addition, suppressing mNAcSh D2 neurons by i.p. injection of CNO increased the sucrose preference (Figure 2G) and reduced immobility (Figure 2, H and I) in SNI 6-week mice. Interestingly, the traveling distances and speeds showed insignificant differences in different groups of mice (Figure 2, J and K), indicating that the alteration of mouse behaviors was not due to a change in locomotor activity. These data suggest that inhibition of mNAcSh D2 neurons can alleviate both pain hypersensitivity and depressive-like behaviors after nerve injury.

Moreover, we examined the behavioral effects of directly activating mNAcSh D2 neurons using chemogenetics. In sham-operated animals, the excitatory DREADD receptor hM3Dq (or mCherry as control) was expressed in the mNAcSh D2 neurons in a Cre-dependent manner (Figure 2, L–N). With slice recording, we verified that hM3Dq-expressing mNAcSh neurons could be activated by perfusion of CNO (Figure 2O). After i.p. administration of CNO, pain hypersensitivity was increased in hM3Dq-expressing mice compared with the vehicle administration and mCherry controls, as demonstrated by the reduced mechanical paw withdrawal threshold and the decreased thermal paw withdrawal latency (Figure 2, P and Q). Activation of mNAcSh D2 neurons in sham mice also enhanced depression-like behaviors (Figure 2, R–T), except for locomotor activity (Figure 2, U and V). These data suggest that activation of mNAcSh D2 neurons is sufficient for the expression of pain hypersensitivity and depression-like behaviors.

### Divergent mNAcSh D2 projections mediate distinct symptoms.

**Figure 3:**
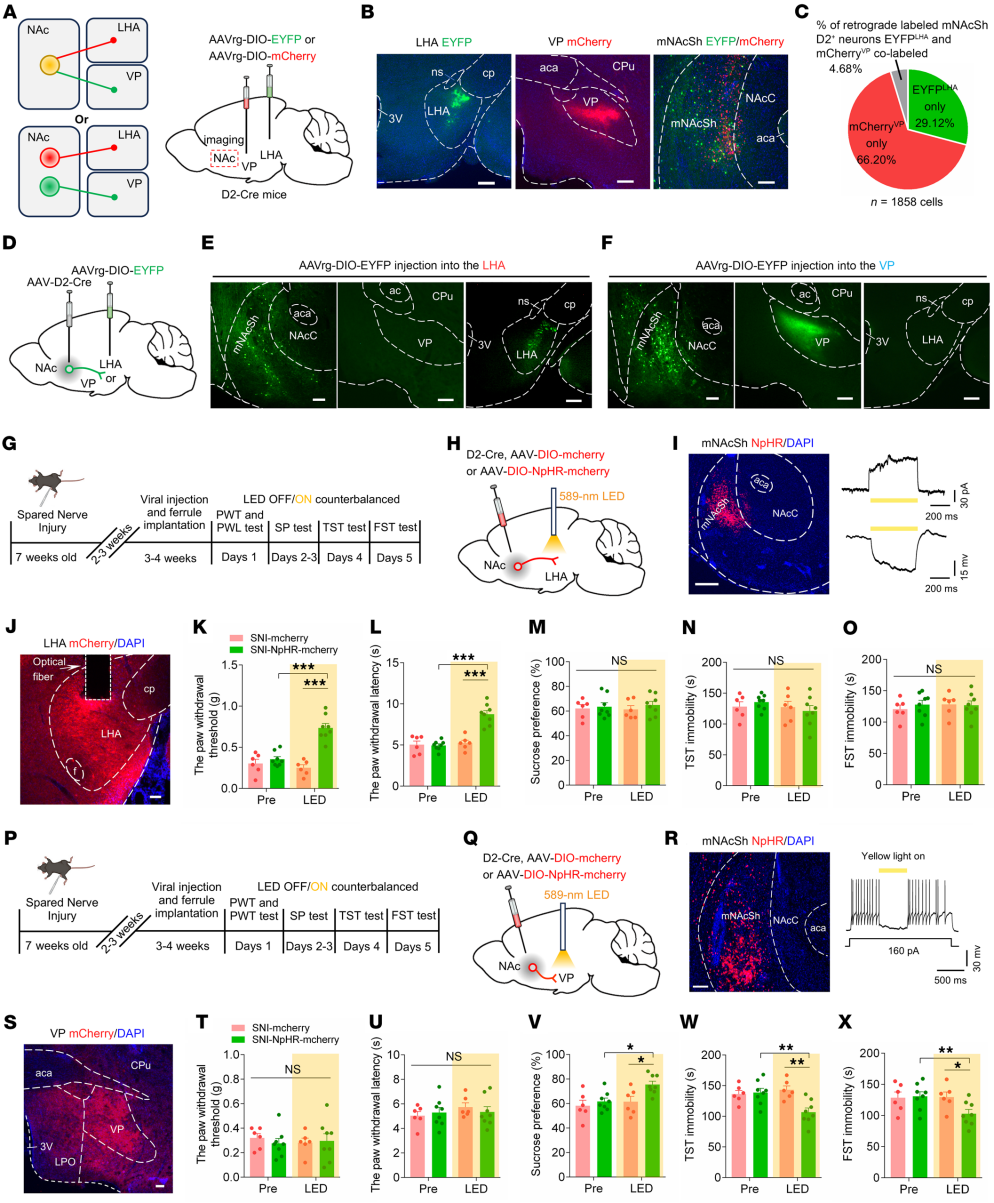
Silencing mNAcSh^D2^-LHA and mNAcSh^D2^-VP projections induces different effects on pain hypersensitivity and depression-like phenotypes in SNI mice. (**A**) Possible projection patterns of mNAcSh^D2^ neurons. (**B** and **C**) Representative images (**B**) and quantitation (**C**) of projection-specific mNAcSh^D2^ neurons. Scale bars: 100 (for NAc) and 200 μm. 3V, 3rd ventricle; ns, nigrostriatal bundle; cp, cerebral peduncle; CPu, caudate putamen. (**D**) Experimental schematic. (**E** and **F**) Representative images of terminals in LHA (**E**) and VP (**F**). Scale bars: 100 (for NAc) and 200 μm. ac, anterior commissure. (**G** and **H**) Experimental timeline (**G**) and schematic (**H**). (**I**) The sample image and recording of NpHR-mCherry-positive neurons. Scale bar, 200 μm. (**J**) Representative image showing mCherry-positive terminals. Scale bar, 100 μm. f, fornix. (**K** and **L**) Mechanical (**K**) and thermal (**L**) pain thresholds (*n* = 6–8 mice/group); interaction *F*_(1, 12)_ = 39.91 (**K**) and *F*_(1, 12)_ = 25.69 (**L**). (**M**–**O**) Sucrose preference (**M**), immobility time in TST (**N**) and FST (**O**) in mice (*n* = 6–8 mice/group); *F*_(1, 12)_ = 0.2476 (**M**); *F*_(1, 12)_ = 1.858 (**N**); and *F*_(1, 12)_ = 0.9139 (**O**). (**P**, **Q**) Experimental timeline (**P**) and schematic (**Q**). (**R**) A sample image and recording of eNpHR-expressing neuron. Scale bar, 100 μm. (**S**) A representative image showing mCherry-positive terminals. Scale bar, 100 μm. (**T** and **U**) Mechanical (**T**) and thermal (**U**) pain thresholds (*n* = 6–8 mice/group); *F*_(1, 12)_ = 0.8222 (**T**) and *F*_(1, 12)_ = 1.077 (**U**). (**V**–**X**) Sucrose preference (**V**), immobility time in TST (**W**), and FST (**X**) (*n* = 6–8 mice/group); *F*_(1, 12)_ = 7.657 (**V**); *F*_(1, 12)_ = 12.19 (**W**) and *F*_(1, 12)_ = 5.973 (**X**). *P < 0.05, **P < 0.01, ***P < 0.001 by 2-way repeated measures ANOVA with Bonferroni’s multiple comparisons test (**K**–**O** and **T**–**X**). Data are represented as mean ± SEM.

How does the activation of mNAcSh D2 neurons result in pain hypersensitivity and depression-like behaviors? Previous studies suggested that NAc divergent projections drive differential motivational processes (38, 39). To identify projection targets of mNAcSh D2 neurons, we labeled mNAcSh D2 axons and presynaptic terminals by expressing mGFP-Synaptophysin-mRuby in a Cre-dependent manner. Abundant fluorescence-labeled axonal projections were mainly in the VP and the LHA (Supplemental Figure 3, A–F). However, it remains unclear whether separate mNAcSh D2 neurons project to VP and LHA or if individual mNAcSh D2 neurons collateralize onto downstream targets (Figure 3A). Therefore, AAVrg-DIO-mCherry and AAVrg-DIO-EYFP were injected into the VP and LHA in D2-Cre mice, respectively (Figure 3, A and B). We found largely nonoverlapping populations of mNAcSh D2 neurons projecting to the VP and LHA (Figure 3C). With confirmed specificity and efficiency (Supplemental Figure 3, G and H), AAV-D2-Cre was injected into the mNAcSh, and AAVrg-DIO-EYFP was injected into the LHA or VP (Figure 3D). Examination of axonal fibers originating from LHA- or VP-projecting mNAcSh D2 neurons (mNAcSh^D2^-LHA and mNAcSh^D2^-VP, respectively) revealed that cells projected primarily to the LHA or VP, but not to both (Figure 3, E and F). Thus, mNAcSh^D2^-LHA and mNAcSh^D2^-VP neurons are anatomically distinct neuronal populations.

We specifically examined the roles of these 2 divergent mNAcSh D2 projections in the expression of pain hypersensitivity and depression-like behaviors. In SNI-treated mice, we expressed Cre-dependent halorhodopsin (eNpHR) in the mNAcSh and optically inhibited the mNAcSh D2 neural axons in the LHA (Figure 3, G–J). Application of yellow light-emitting diode (LED) light increased the mechanical paw withdrawal threshold and the thermal paw withdrawal latency (Figure 3, K and L) compared with the LED-off condition and with mCherry control animals in the same light application condition. However, silencing the mNAcSh D2 projection to LHA did not change the sucrose preference, the immobility time in the TST, and the immobility time in the FST (Figure 3, M–O). To verify the involvement of the mNAcSh^D2^-LHA neurons in pain-related behaviors, in sham-operated mice, we stimulated the mNAcSh D2 axon terminals in the LHA (Supplemental Figure 4, A–C). Compared with mCherry controls, activation of the mNAcSh^D2^-LHA pathway resulted in pain hypersensitivity, yet did not affect sucrose preference and immobility behaviors in mice (Supplemental Figure 4, D–G). These data suggested that the mNAcSh D2 projection to LHA may selectively mediate the pain-related behaviors but not the depression-like symptoms.

We further tested the role of mNAcSh^D2^-VP projection in the modulation of pain-related behaviors and depression-like phenotypes. In the SNI-treated mice, eNpHR was Cre-dependently expressed in the mNAcSh D2 neurons, and neural terminals in the VP were optically inhibited (Figure 3, P–S). Silencing the mNAcSh^D2^-VP pathway increased the sucrose preference and decreased immobility time while not affecting pain-related behaviors (Figure 3, T–X). Conversely, we optogenetically activated the mNAcSh^D2^-VP pathway in sham-operated mice (Supplemental Figure 4, H–J). Behavioral data revealed that activating this pathway decreased the sucrose preference and increased immobility time, with the pain threshold intact in mice (Supplemental Figure 4, K–N). These data suggested that the alleviating effect of silencing mNAcSh D2 neurons could be attributed to their projections to the LHA and VP and that divergent mNAcSh D2 neural circuits preferably contributed to pain hypersensitivity and comorbid depression-like behaviors.

### mNAcSh D2 neurons mediated chronic pain via innervating the LHA orexin neurons.

**Figure 4:**
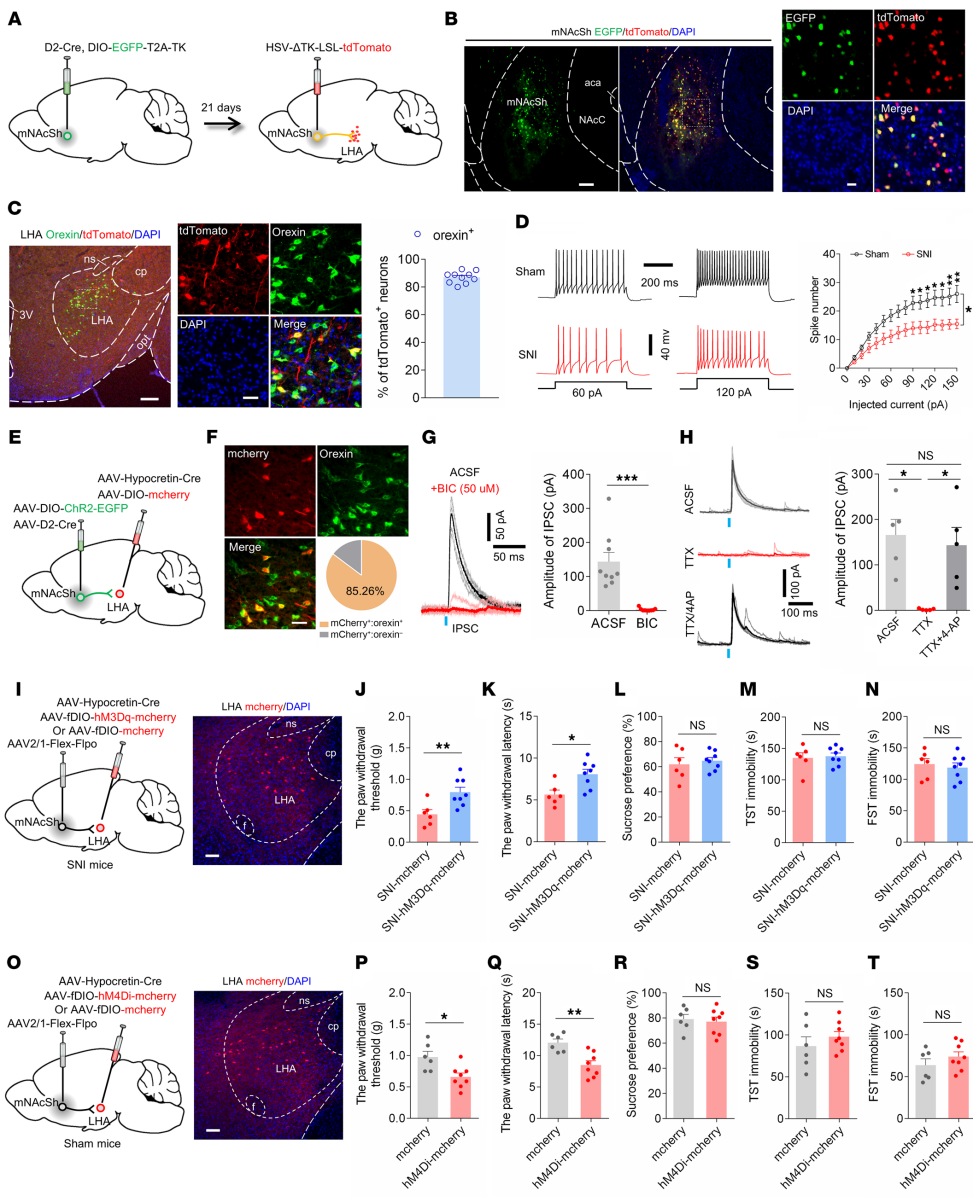
The mNAcSh^D2^-LHA^orexin^ pathway mediates nerve injury–induced pain hypersensitivity. (**A**) Illustration showing virus injection. (**B**) Representative images show tdTomato expression in mNAcSh D2 cells (green). Scale bars: 100 μm (left), 20 μm (right). (**C**) Representative images and plot reveal LHA cells (red) labeled with anti-orexin (green). Scale bars: 200 μm (left) and 30 μm (right). (**D**) Representative traces and input-output function of postsynaptic LHA neurons in mice (*n* = 12 and 14 cells); *F*_(15,__360)_ = 5.176. (**E**) Scheme for virus injection. (**F**) Representative images and plot reveal mCherry-positive LHA neurons labeled with anti-orexin (green). Scale bar: 30 μm. (**G**) Light-evoked IPSC was blocked by bicuculline (*n* = 9 neurons). Paired *t* test, t_8_ = 5.119, ***P = 0.0009. (**H**) Optical IPSC was blocked by TTX and recovered by TTX/4-AP (*n* = 5 neurons); *F*_(1.057, 4.229)_ = 16.25. (**I**) Scheme and representative image showing infection of LHA neurons with hM3Dq-mCherry. Scale bar: 100 μm. (**J** and **K**) Mechanical (**J**) and thermal (**K**) pain thresholds in SNI mice (*n* = 6–8 mice/group); t_12_ = 3.135 (**J**); t_12_ = 2.989 (**K**). (**L**–**N**) Sucrose preference (**L**) and immobility time in TST (**M**) and FST (**N**) (*n* = 6–8 mice/group); t_12_ = 0.5172 (**L**); t_12_ = 0.2910 (**M**); t_12_ = 0.4693 (**N**). (**O**) Scheme and representative image showing specific infection of LHA neurons with hM4Di-mCherry. Scale bar: 100 μm. (**P** and **Q**) Mechanical (**P**) and thermal (**Q**) pain thresholds in sham-operated mice (*n* = 6–8 mice); t_12_ = 2.987 (**P**); t_12_ = 3.791 (**Q**). (**R**–**T**) Sucrose preference (**R**) and immobility time in TST (**S**) and FST (**T**) (*n* = 6–8 mice); t_12_ = 0.3659 (**R**); t_12_ = 0.9411 (**S**); t_12_ = 1.081 (**T**). *P < 0.05 and **P < 0.01 by 1-way (**H**) or 2-way (**D**) repeated-measure ANOVA with Bonferroni’s multiple-comparison test or by 2-tailed *t* test (**J**–**N** and **P**–**T**). Data are represented as mean ± SEM.

The LHA has been documented to contribute to pain-related behaviors (40, 41). To investigate how mNAcSh D2 neurons might innervate the LHA, we detected the synaptic targets of mNAcSh D2 neurons using genetically modified virus tracing tools (42). First, thymidine kinase (TK) was Cre-dependently expressed in mNAcSh D2 neurons. We injected a genetically modified version of Herpes simplex virus type 1 (HSV-ΔTK-LSL-tdTomato) in which TK was deleted (Figure 4A). This resulted in the expression of EGFP and tdTomato in mNAcSh D2 neurons (Figure 4B) and expression of tdTomato in postsynaptic LHA neurons (Figure 4C). Immunofluorescence staining indicated that a majority of postsynaptic LHA neurons expressed orexin (Figure 4C). Moreover, these postsynaptic LHA orexinergic neurons showed reduced membrane excitability in SNI 6-week mice compared with sham-operated mice (Figure 4D), which was partially consistent with a previous study reporting inhibition of orexin neurons in response to noxious stimuli (40).

To further test how mNAcSh D2 neurons innervate LHA orexinergic neurons, we injected AAV-DIO-ChR2-EGFP and AAV-D2-Cre into the mNAcSh, with AAV-Hypocretin-Cre and AAV-DIO-mCherry injected into the LHA (Figure 4E). We found that blue light evoked exclusively inhibitory postsynaptic currents (IPSCs) in the mCherry-positive LHA orexinergic neurons (Figure 4F), which could be blocked by the GABA_A_ receptor antagonist bicuculline (Figure 4G). Furthermore, the recorded postsynaptic currents were completely blocked by the application of tetrodotoxin (TTX) and recovered by the application of TTX and 4-Aminopyridine (4-AP) (Figure 4H), indicating that the postsynaptic currents were elicited by direct synaptic connections between LHA-projecting NAc D2 neurons and the recorded LHA orexinergic neurons (12).

The modulatory roles of manipulating the mNAcSh^D2^-LHA neurons in pain sensation might be due to altered functions in axon collaterals. Additionally, although the orexin system is involved in pain modulation (43), the neural circuit mechanisms remain unclear. We therefore injected AAV2/1-Flex-Flpo into the mNAcSh and infected LHA postsynaptic orexinergic neurons with a Cre-dependent virus encoding the neuronal excitatory DREADD hM3Dq (AAV2/9-fDIO-hM3Dq-mCherry) (Figure 4I). We found that specific activation of LHA postsynaptic orexinergic neurons decreased pain hypersensitivity but not the depression-like behaviors in SNI mice (Figure 4, J–N). Conversely, inhibitory DREADD hM4Di (AAV2/9-fDIO-hM4Di-mCherry) was selectively expressed in LHA postsynaptic orexinergic neurons in sham mice (Figure 4O). Behavioral data demonstrated that chemogenetic inhibition of the postsynaptic orexinergic neurons of the mNAcSh notably decreased the pain threshold (Figure 4, P and Q), but not the depression-like behaviors (Figure 4, R–T), in sham-operated mice. Together, these observations indicated that mNAcSh D2 neurons preferably innervated LHA orexinergic neurons and contributed to chronic pain modulation.

### mNAcSh D2 neurons mediate depression-like behaviors by disinhibiting VP glutamatergic neurons.

**Figure 5:**
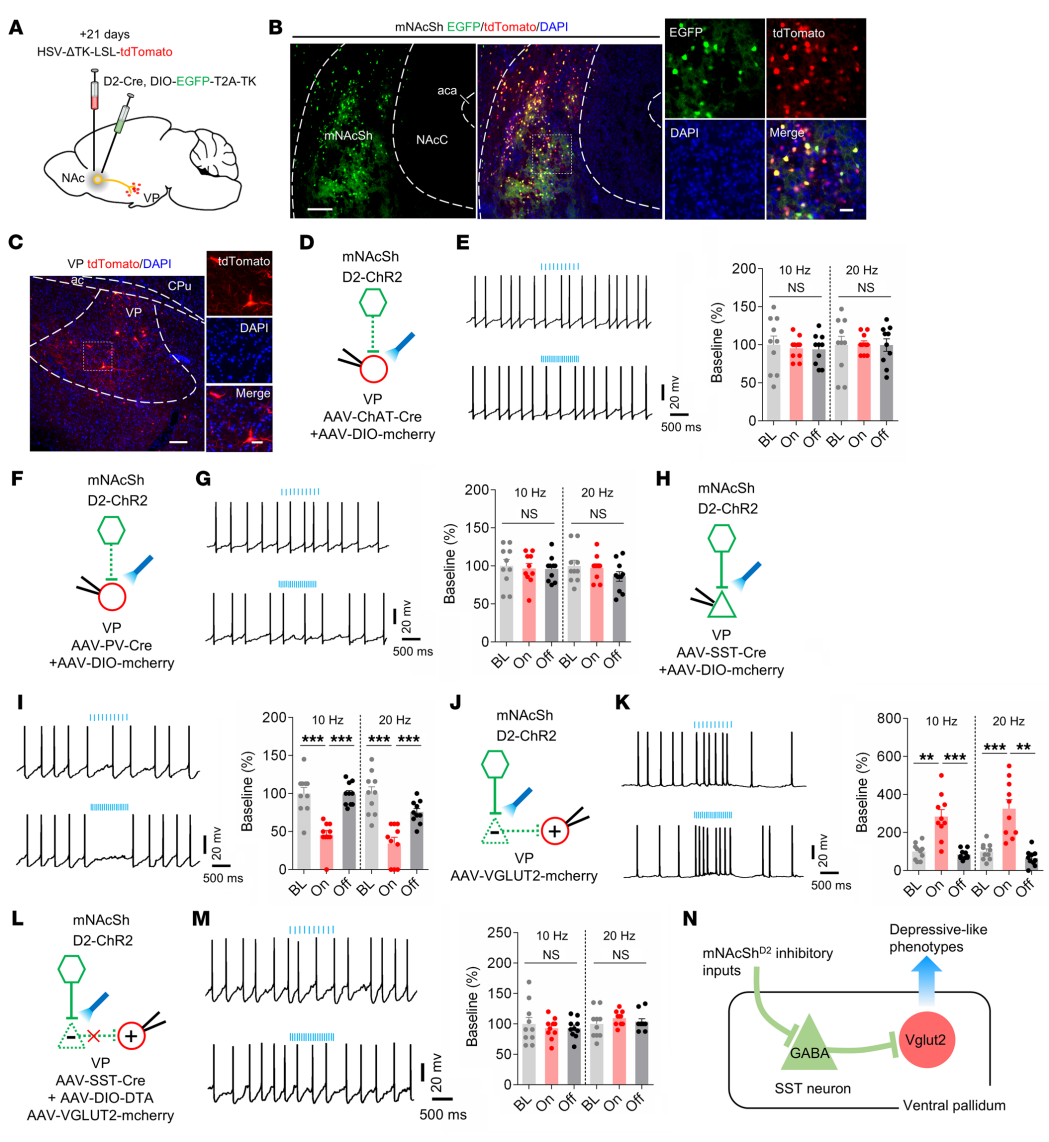
mNAcSh D2 inputs disinhibit VP glutamatergic neurons via inhibition of SST neurons. (**A**) Schematic showing HSV-based anterograde tracing. (**B**) Representative images show tdTomato expression in mNAcSh D2 neurons (green). Scale bars: 100 μm (left), 20 μm (right). (**C**) Sample images reveal postsynaptic VP cells. Scale bars: 100 μm (left), 25 μm (right). (**D**, **F**, and **H**) Schematics of recordings in VP ChAT-mCherry–positive (**D**), PV-mCherry–positive (**F**), and SST-mCherry–positive (**H**) neurons. (**E**, **G**, and **I**) Sample traces (left) and bar plots (right) showing the effects of photoactivating mNAcSh D2 inputs on firing rates of VP ChAT^+^ neurons (**E**) [*n* = 10 neurons; 10 Hz: *F*_(1.704, 15.34)_ = 0.3289; 20 Hz: *F*_(1.509, 13.58)_ = 0.006729], PV^+^ neurons (**G**) [*n* = 10 neurons; 10 Hz: *F*_(1.541, 13.87)_ = 0.07384; 20 Hz: *F*_(1.569, 14.12)_ = 1.297], and SST^+^ neurons (**I**) [*n* = 10 neurons; 10 Hz: *F*_(1.797, 16.17)_ = 29.65; 20 Hz: *F*_(1.696, 15.27)_ = 21.93]. (**J**) Schematics showing recording of mCherry-positive VP glutamatergic neurons. Green triangle indicates inhibitory VP neurons, presumably receiving input from mNAcSh and innervating VP glutamatergic neurons. (**K**) Sample traces (left) and bar plots (right) showing firing rates of VP glutamatergic neurons (*n* = 10 neurons); 10 Hz: *F*_(1.397, 12.57)_ = 25.53; 20 Hz: *F*_(1.270, 11.43)_ = 24.80. (**L**) Schematics of recordings in VP glutamatergic neurons during photoactivating D2 terminals with ablation of SST^+^ neurons. (**M**) The effects of ablating VP SST^+^ neurons on the firing rates of VP glutamatergic neurons (*n* = 10 neurons); 10 Hz: *F*_(1.336, 12.02)_ = 0.3194; 20 Hz: *F*_(1.533, 13.79)_ = 0.6932. (**N**) Putative VP microcircuits. Increased inhibitory inputs onto VP SST neurons cause a disinhibition of VP glutamatergic neurons, thereby enhancing depression-like behavior. **P < 0.01 and ***P < 0.001 by 1-way repeated-measure ANOVA with Bonferroni’s multiple-comparison test (**E**, **G**, **I**, **K**, and **M**). Data are represented as mean ± SEM.

Since mNAcSh D2 neural axons terminated in the VP, which has been implicated in integrating and transmitting depression- and pain-relevant signals across the brain (5, 44), we examined how mNAcSh D2 neurons innervated VP neurons. mNAcSh D2 neurons were Cre-dependently infected with AAV-DIO-EGFP-T2A-TK, and HSV-ΔTK-LSL-tdTomato was injected into the mNAcSh 3 weeks later (Figure 5A). Only mNAcSh neurons expressing TK allowed the antegrade spreading of HSV to postsynaptic VP neurons (output cells) (Figure 5, B and C). The VP exhibits considerable cellular heterogeneity and is mainly composed of glutamatergic neurons, cholinergic neurons, and somatostatin-positive (SST^+^) and parvalbumin-positive (PV^+^) GABAergic neurons (44, 45). To target different VP cell types, we used AAVs expressing vesicular glutamate transporter 2–Cre (VGLUT2-Cre), choline acetyltransferase–Cre (ChAT-Cre), SST-Cre, and PV-Cre for cell type–specific expression of GFP. Meanwhile, postsynaptic VP neurons innervated by the mNAcSh D2 neurons were labeled with tdTomato using HSV-mediated antegrade tracing (Supplemental Figure 5A). We found the most extensive expression of HSV-tdTomato^+^ in SST^+^ neurons (Supplemental Figure 5, B and C), rather than other genetically defined cell types (Supplemental Figure 5, D–I). These data indicated that mNAcSh D2 neurons mainly synapse onto SST^+^ neurons within the VP.

Next, we examined whether and how mNAcSh D2 neurons modulate the different subpopulations of VP neurons. Hence, during photoactivation of ChR2-expressing mNAcSh D2 neural terminals in slices, we recorded the firing rates of the genetically specified VP neurons labeled with mCherry. When the mNAcSh^D2^-VP circuit was photoactivated, neither ChAT^+^ (Figure 5, D and E) nor PV^+^ (Figure 5, F and G) neurons revealed changes in the firing rates. Interestingly, blue light illumination inhibited the firing rates of SST^+^ neurons (Figure 5, H and I). In contrast, the firing rates of VGLUT2^+^ VP neurons were increased when mNAcSh D2 terminals were photoactivated (Figure 5, J and K). Based on these results, we hypothesized that activation of mNAcSh D2 neurons could disinhibit VP glutamatergic neurons by directly inhibiting local SST^+^ interneurons. To further test this disinhibition model, VP SST^+^ neurons were ablated through Cre recombinase–mediated expression of diphtheria toxin subunit A (DTA) in mice (Figure 5L). We found that selective ablation of VP SST^+^ neurons blocked the activation of VP glutamatergic neurons by photoactivation of mNAcSh D2 terminals (Figure 5M).

VP glutamatergic neurons participate in the modulation of emotion-related behaviors (44, 46). We assumed that mNAcSh D2 neurons mediate depression-like phenotypes by disinhibiting VP glutamatergic. To test this hypothesis, the excitability of VP glutamatergic neurons was tested using patch-clamp recording (Supplemental Figure 6A). We found that the VP glutamatergic neurons were activated in SNI mice comorbid with depression (Supplemental Figure 6, B and C). Next, hM4Di and ChR2 were Cre-dependently expressed in the mNAcSh D2 neurons and VP glutamatergic neurons, respectively (Supplemental Figure 6D). Behavioral data showed that chemogenetic silencing of mNAcSh D2 neurons significantly attenuated the depression-like behaviors in nerve-injured mice, which was reversed by photoactivation of VP glutamatergic neurons (Supplemental Figure 6, G and H). In contrast, optogenetic activation of VP glutamatergic neurons produced insignificant changes in the analgesic effects induced by chemogenetic silencing of mNAcSh D2 neurons (Supplemental Figure 6, E and F). Additionally, VP SST^+^ neurons were ablated through Cre-dependent expression of DTA in SNI mice (Supplemental Figure 6, I–K). In these mice, chemogenetic silencing of mNAcSh D2 neurons could not relieve the depression-like behaviors (Supplemental Figure 6, L–O). Collectively, these results suggest that mNAcSh D2 neurons cause disinhibition of VP glutamatergic neurons, thereby resulting in depression-like behavior (Figure 5N)

### Upstream connectivity of mNAcSh^D2^-LHA and mNAcSh^D2^-VP neurons.

We next estimated the upstream connectivity of divergent mNAcSh D2 subpopulations by first measuring the miniature excitatory postsynaptic current and inhibatory postsynaptic current (mEPSC and mIPSC, respectively) in these neurons. We found an increase in the frequency and amplitude of mEPSC in mNAcSh^D2^-LHA neurons in nerve-injured mice, yet no changes in mIPSC (Supplemental Figure 7, A–G), indicating enhanced excitatory drive onto mNAcSh^D2^-LHA neurons. We additionally found a decrease in the frequency of mIPSC, but not mEPSC, in mNAcSh^D2^-VP neurons from SNI mice (Supplemental Figure 7, H–N). Interestingly, although both populations exhibit enhanced excitability, the synaptic adaptations underlying this alteration differ.

**Figure 6:**
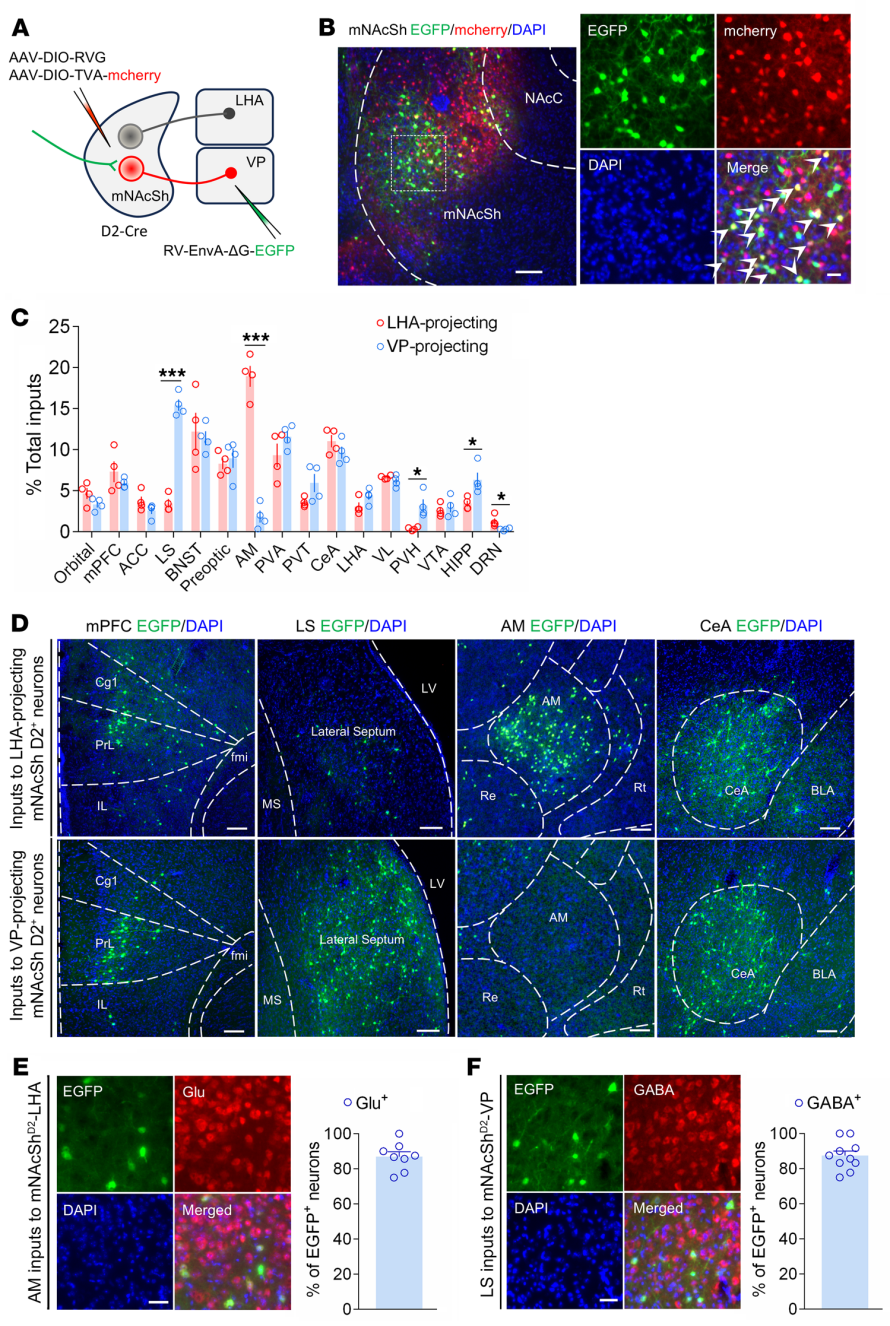
Whole-brain mapping of inputs to mNAcSh^D2^-LHA and mNAcSh^D2^-VP neurons. (**A**) Injection regimen to map pseudotype rabies–mediated monosynaptic inputs to either LHA- or VP-projecting mNAcSh D2 neurons. (**B**) Starter cell localization of AAV-DIO-TVA-mCherry and RV-EnvA-ΔG-EGFP (bottom, white arrowheads). Scale bars: 200 μm (left), 20 μm (right). (**C**) Whole-brain quantitation of inputs to mNAcSh^D2^-LHA and mNAcSh^D2^-VP neurons (*n* = 4 for each condition). Unpaired *t* test was used for individual brain regions; *P < 0.05 and ***P < 0.001. (**D**) Representative images of inputs in select brain areas. Scale bars: 100 μm. Cg1, cingulate cortex, area 1; PrL, prelimbic cortex; IL, infralimbic cortex; fmi, forceps minor of the corpus callosum; MS, medial septal nucleus; LV, lateral ventricle; Re, reuniens thalamic nucleus; Rt, reticular thalamic nucleus; BLA, basolateral amygdaloid nucleus. (**E**) Representative images and quantitation of the mNAcSh^D2^-LHA–projecting AM neurons colocalized with glutamate immunofluorescence (*n* = 8). Scale bar: 25 μm. (**F**) Sample images (left) and percentage (right) of the mNAcSh^D2^-VP–projecting LS neurons colocalized with GABA immunofluorescence (*n* = 10). Scale bar: 25 μm. Data are represented as mean ± SEM.

Since mNAcSh^D2^-LHA and mNAcSh^D2^-VP neurons can be distinguished based on their efferent target, we hypothesized that they may also receive distinct inputs. To test this, we probed the input-output organization of mNAcSh D2 neurons by cell type and projection-specific transsynaptic tracing (14). First, we injected Cre-dependent AAVs expressing TVA receptor and the rabies glycoprotein into mNAcSh (AAV-DIO-TVA-mCherry and AAV-DIO-RVG, respectively) of D2-Cre mice. We then delivered EnvA (envelope derived from avian sarcoma leukosis virus subtype A)-pseudotyped, glycoprotein-deleted rabies virus (RV-EnvA-ΔG-EGFP) into the LHA or VP (Figure 6A). The starter neurons (EGFP and mCherry double-stained) were localized in the mNAcSh (Figure 6B). Whole-brain quantitation of EGFP-labeled neurons revealed differences in inputs to mNAcSh^D2^-LHA and mNAcSh^D2^-VP neuronal populations. In particular, while mNAcSh^D2^-LHA neurons received proportionally more input from the AM, mNAcSh^D2^-VP neurons received more input from the LS (Figure 6, C and D).

Considering that the AM-to-NAc and LS-to-NAc connections have been poorly defined, we combined input-output viral tracing with immunofluorescence staining to investigate the molecular identity of AM and LS input neurons, respectively. We found the most extensive colocalization of rabies-EGFP^+^ AM neurons with glutamate (Figure 6E). However, quantitation revealed that mNAcSh^D2^-VP neurons received input from GABA-expressing LS neurons (Figure 6F). Together, these data demonstrate that mNAcSh^D2^-LHA and mNAcSh^D2^-VP neurons receive excitatory or inhibitory inputs from distinct brain areas.

### AM glutamatergic inputs to mNAcSh activate LHA-projecting D2 neurons and promote pain hypersensitivity.

**Figure 7:**
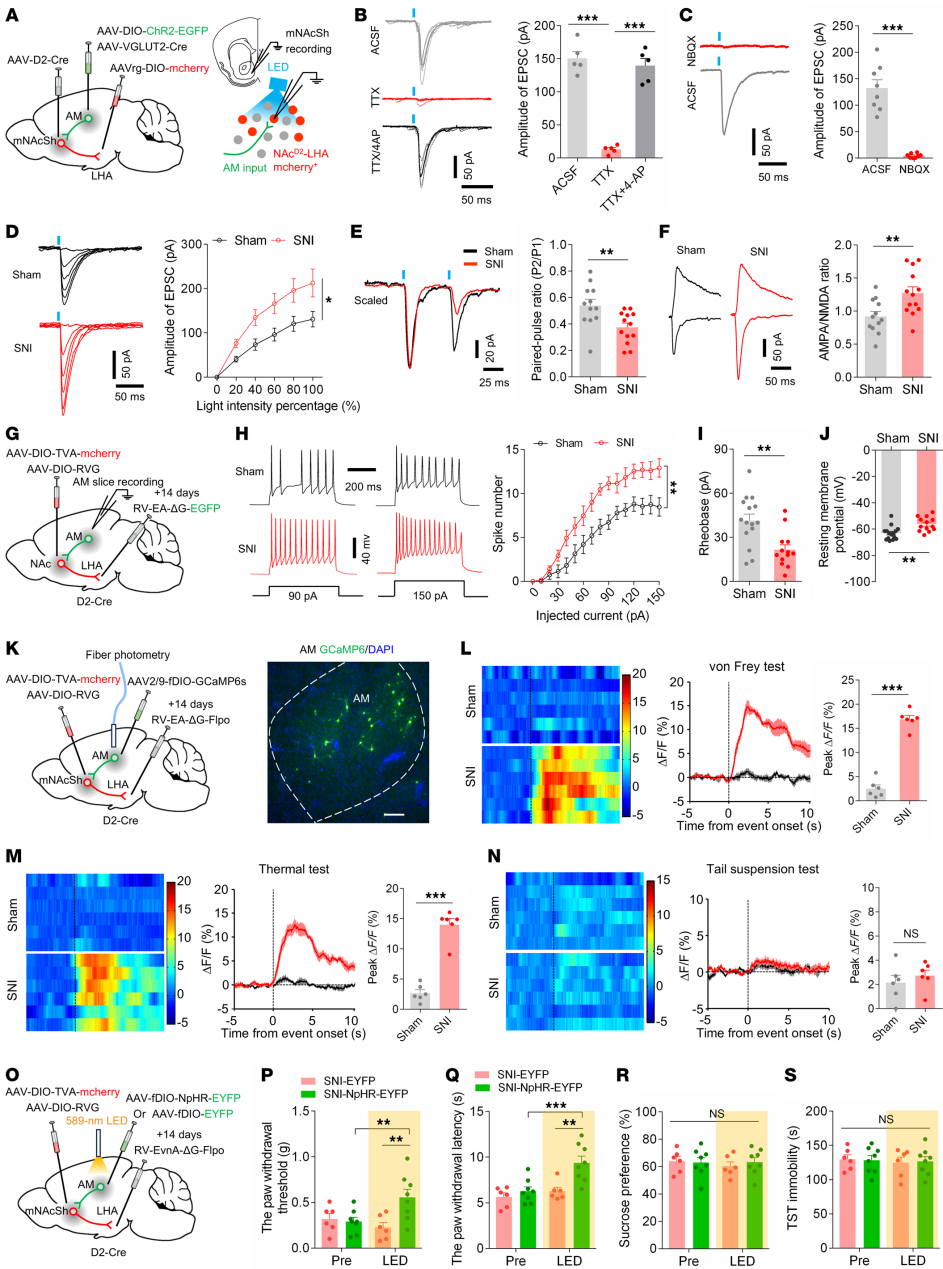
The AM^Glu^-mNAcSh^D2^-LHA^orexin^ pathway controls pain hypersensitivity. (**A**) Schematic showing viral injection. (**B**) Light-evoked currents were blocked by TTX and restored by TTX+4-AP (*n* = 5); *F*_(1.749, 6.994)_ = 119.6. (**C**) NBQX blocked light-evoked currents. Paired *t* test; t_7_ = 8.435, ***P < 0.001. (**D**) Examples and input-output curves of EPSC evoked by a progressive increase in light intensity (*n* = 10-11 neurons/group); *F*_(1, 19)_ = 6.340. (**E**) Representative traces and statistics showing PPR of EPSC (*n* = 12-13 neurons); t_23_ = 2.956. (**F**) Sample traces and statistics of AMPA/NMDA ratio of EPSC (*n* = 12–13 neurons); t_23_ = 2.934. (**G**) Schematic showing viral injection. (**H**) The membrane excitability of AM-mNAcSh^D2^-LHA neurons (*n* = 13–15 neurons); *F*_(15, 390)_ = 2.896. (**I** and **J**) The rheobase (**I**) and RMP (**J**) of AM-mNAcSh^D2^-LHA neurons (*n* = 13–15 neurons); t_26_ = 3.475, (**I**); t_26_ = 3.470 (**J**). (**K**) Illustration and representative image showing viral injection. Scale bar, 100 μm. (**L**–**N**) Heat maps, averaged responses, and peak Δ*F*/*F* of the Ca^2+^ signals during von Frey (**L**), thermal stimuli (**M**), or tail suspension (**N**) (*n* = 6 animals/group); t_10_ = 13.16 (**L**); t_10_ = 9.728 (**M**); t_10_ = 0.7716 (**N**). (**O**) Experimental schematic. (**P** and **Q**) Mechanical (**P**) and thermal (**Q**) pain thresholds in SNI mice (*n* = 6–8 mice/group); *F*_(1, 12)_ = 12.75, (**P**); *F*_(1, 12)_ = 6.763 (**Q**). (**R** and **S**) Sucrose preference (**R**) and immobility time in TST (**S**) in mice (*n* = 6–8 mice/group); *F*_(1, 12)_ = 0.2662 (**R**); *F*_(1, 12)_ = 0.05487 (**S**). *P < 0.05, **P < 0.01, ***P < 0.001 by 1-way (**B**) or 2-way repeated measures ANOVA with Bonferroni’s multiple comparisons test (**D**, **H** and **P**–**S**). **P < 0.01, ***P < 0.001 by 2-tailed *t* test (**E**, **F**, **I**, **J**, and **L**–**N**). Data are represented as mean ± SEM.

To examine how the AM neurons make functional synaptic connections onto mNAcSh^D2^-LHA neurons, we combined retrograde tracing and ex vivo electrophysiology (Figure 7A). Notably, following Cre-dependent expression of ChR2 in glutamatergic (i.e., VGLUT2-expressing) AM neurons, blue light stimulation of AM terminals produced monosynaptic EPSCs in mNAcSh^D2^-LHA neurons (Figure 7B). Light-evoked EPSCs were blocked by an α-amino-3-hydroxy-5-methyl-4-isoxazole-propionate (AMPA) receptor antagonist, 2,3-dihydroxy-6-nitro-7-sulphamoyl-benzo (F)quinoxaline (5 μM NBQX), indicating that AM terminals released glutamate (Figure 7C). We found that the amplitude of EPSC was increased at various light intensities, and the EPSC paired-pulse ratio (PPR) was decreased in mNAcSh^D2^-LHA neurons in SNI mice comorbid with depression (Figure 7, D and E), indicating enhanced presynaptic glutamate release in these mice. Furthermore, the ratio of light-evoked AMPA receptor– and NMDA receptor–mediated EPSC (AMPA/NMDA ratio) in mNAcSh^D2^-LHA neurons was markedly increased in nerve-injured mice (Figure 7F). Overall, these results demonstrated a direct functional connection and synaptic plasticity in the AM^Glu^-mNAcSh^D2^ circuit.

Next, we wondered whether presynaptic AM neurons innervating the mNAcSh^D2^-LHA (hence defined as AM^Glu^-mNAcSh^D2^-LHA neurons) could respond to chronic pain with excitability changes. We labeled the AM^Glu^-mNAcSh^D2^-LHA neurons with rabies virus and carried out whole-cell patch-clamp recording in brain slices (Figure 7G). Selective recording from rabies-EGFP–labeled AM neurons revealed significantly increased firing rates, reduced spike threshold, and lower resting membrane potential in nerve-injured mice (Figure 7, H–J). Next, we used fiber photometry to measure the stimuli-induced (von Frey filament, thermal stimuli, and tail suspension) changes in Ca^2+^ signals in AM^Glu^-mNAcSh^D2^-LHA neurons (Figure 7K). We found that pain-related stimuli reliably evoked a robust activation of AM^Glu^-mNAcSh^D2^-LHA neurons in SNI mice (Figure 7, L and M). However, no effect was observed on AM^Glu^-mNAcSh^D2^-LHA neurons during depression-related stimulation (Figure 7N). These results suggest that nerve injury results in activation of specific AM neurons projecting to the mNAcSh^D2^-LHA neurons.

It has been demonstrated that the thalamus plays a pivotal role in the regulation of nocifensive behaviors (47), yet the role of AM in pain modulation is unknown. This prompted us to explore the influence of the AM^Glu^-mNAcSh^D2^-LHA pathway on pain or depression-like behaviors. We first tested the effects of AM^Glu^-mNAcSh^D2^-LHA neuron inhibition on pain hypersensitivity and comorbid depression in SNI mice. We selectively expressed the rabies virus–based Flp recombinase in presynaptic AM neurons of mNAcSh^D2^-LHA neurons and delivered the AAV-fDIO-NpHR-EYFP (or AAV-fDIO-EYFP as control) into the AM (Figure 7O). Optogenetic inhibition of AM^Glu^-mNAcSh^D2^-LHA neurons was sufficient to increase the mechanical and thermal pain threshold in SNI mice (Figure 7, P and Q). Nevertheless, no effect was observed on depression-like behaviors during the inhibition of AM^Glu^-mNAcSh^D2^-LHA neurons in nerve-injured mice (Figure 7, R and S). Thus, suppressing AM^Glu^-mNAcSh^D2^-LHA neurons could ameliorate pain hypersensitivity in mice.

To rule out the possibility that the modulatory roles of the AM inputs to mNAcSh^D2^ in pain behaviors might involve other (parallel) pathways, we directly activated the AM^Glu^-mNAcSh^D2^-LHA neurons using optogenetics and activated LHA orexinergic neurons with chemogenetics in sham animals (Supplemental Figure 8, A–C). The application of blue LED light reduced the mechanical paw withdrawal threshold and thermal paw withdrawal latency compared with the EYFP control. Chemogenetic activation of LHA orexinergic neurons prevented the algesic effects induced by photoactivation of AM^Glu^-mNAcSh^D2^-LHA neurons (Supplemental Figure 8, D and E). However, the depression-like behaviors were similar in different groups of mice (Supplemental Figure 8, F–H). Taken together, these results identify a long-range AM^Glu^-mNAcSh^D2^-LHA^orexin^ circuit that mediates pain response modulation.

### The LS is the input of the mNAcSh^D2^-VP pathway for the expression of depression-like phenotypes in mice with chronic pain.

**Figure 8:**
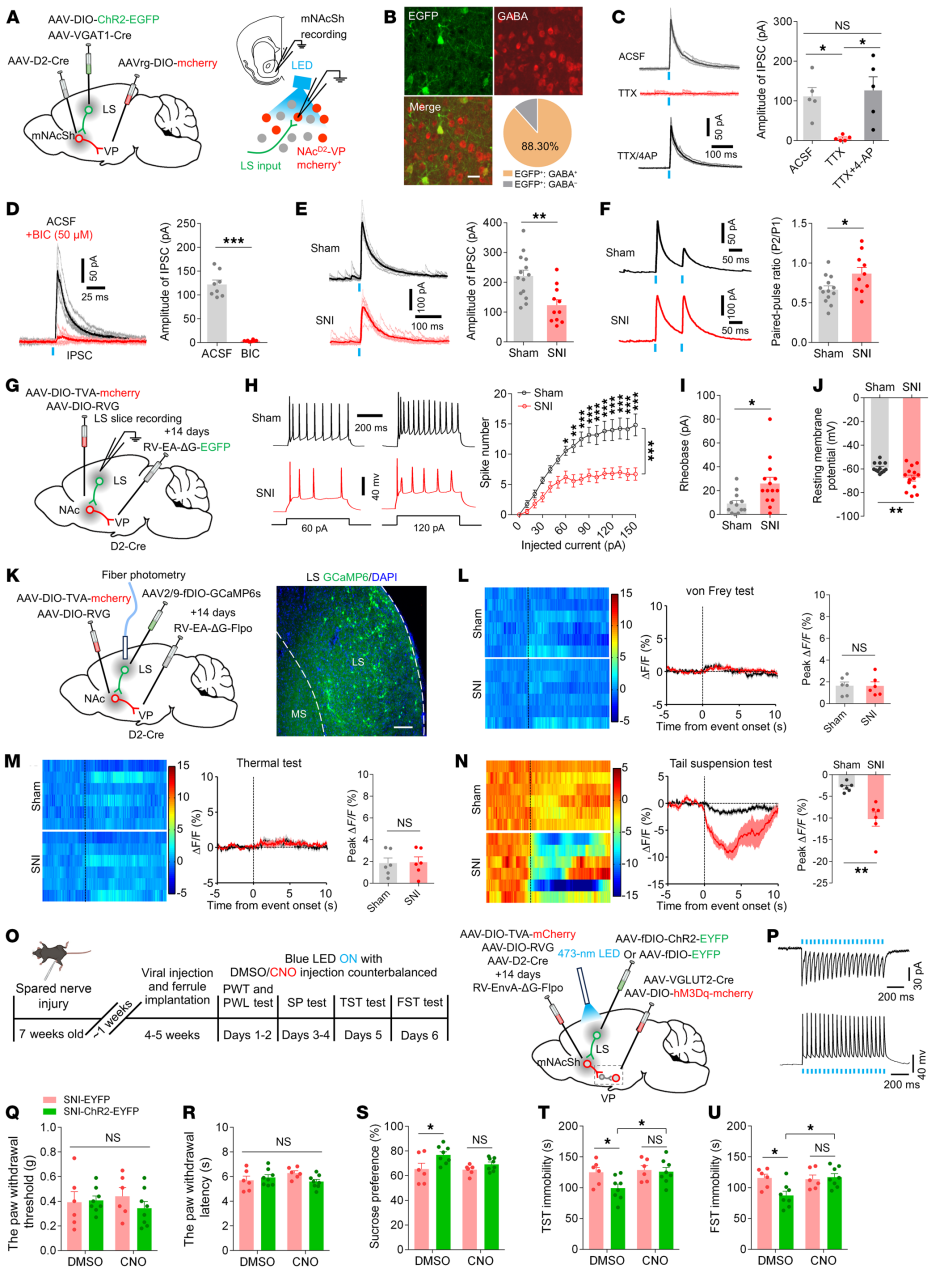
LS GABAergic neurons modulate depression-like behaviors by connection with the mNAcSh^D2^-VP pathway. (**A**) Experimental schematic. (**B**) Percentage of VGAT1-positive neurons labeled with anti-GABA. Scale bar, 20 μm. (**C**) Light-evoked currents were blocked by TTX and recovered by TTX+4-AP (*n* = 5); *F*_(1.695, 6.781)_ = 10.91. (**D**) Bicuculline blocked light-evoked currents; t_7_ = 12.68. (**E**) Representative traces and comparison of IPSC amplitude (*n* = 11–14 neurons); t_23_ = 3.536. (**F**) Sample traces and statistics of IPSC PPR (*n* = 10–13 neurons); t_21_ = 2.312. (**G**) Experimental schematic. (**H**) The membrane excitability of LS-mNAcSh^D2^-VP neurons in mice (*n* = 12–14 neurons); *F*_(15, 360)_ = 6.809, ***P < 0.001. (**I** and **J**) The rheobase (**I**) and RMP (**J**) of LS-mNAcSh^D2^-VP neurons (*n* = 12–14 neurons); t_24_ = 2.681 (**I**); t_24_ = 2.886 (**J**). (**K**) Schematic and representative image showing GCaMP6s expression. Scale bar, 100 μm. (**L**–**N**) Heat maps, averaged responses, and peak Δ*F*/*F* of Ca^2+^ signals upon to von Frey (**L**), thermal stimuli (**M**), or tail suspension (**N**) (*n* = 6 animals/group); t_10_ = 0.02689 (**L**); t_10_ = 0.1267 (**M**); t_10_ = 4.277 (**N**). (**O**) Experimental timeline and scheme. (**P**) Representative traces of ChR2-expressing neurons with photostimulation. (**Q** and **R**) Mechanical (**Q**) and thermal (**R**) pain thresholds in mice (*n* = 6–8 mice/group); *F*_(1, 12)_ = 0.6986 (**Q**); *F*_(1, 12)_ = 1.179 (**R**). (**S**–**U**) Sucrose preference (**S**), immobility time in TST (**T**) and FST (**U**) in mice (*n* = 6–8 mice/group); *F*_(1, 12)_ = 6.905 (**S**); *F*_(1, 12)_ = 4.847 (**T**); *F*_(1, 12)_ = 4.898 (**U**). *P < 0.05, **P < 0.01, ***P < 0.001 by 1-way (**C**) or 2-way repeated-measures ANOVA with Bonferroni’s multiple comparisons test (**H**, **Q**–**U**). *P < 0.05, **P < 0.01 by 2-tailed *t* test (**E**, **F**, **I**, **J**, **L**–**N**). Data are represented as mean ± SEM.

The LS regulates emotional processes and participates in the pharmacological actions of antidepressant drugs (48–50), yet little is known regarding its neurocircuitry basis. Since the LS GABAergic neurons act as a prominent presynaptic partner of the VP-projecting mNAcSh D2 neurons (Figure 6), we investigated how this disynaptic pathway (hence defined as LS^GABA^-mNAcSh^D2^-VP) contributed to comorbid depression in nerve-injured mice. We selectively recorded from mNAcSh^D2^-VP neurons in slice while optogenetically activating ChR2-expressing axons from the LS GABAergic neurons (Figure 8, A and B). Light-evoked monosynaptic IPSC was observed, which could be blocked by a GABA_A_ receptor antagonist, bicuculline (Figure 8, C and D). Furthermore, we demonstrated a reduction in the amplitude and an increment in the PPR in light-evoked IPSC from SNI mice (Figure 8, E and F), indicating decreased inhibitory synapses from LS GABAergic neurons onto the mNAcSh^D2^-VP pathway.

We next analyzed the excitability of LS^GABA^-mNAcSh^D2^-VP neurons by conducting whole-cell recordings (Figure 8G). LS EGFP^+^ neurons displayed a lower excitability in response to depolarizing current steps, an increased rheobase, and a decreased resting membrane potential (RMP) in SNI mice, indicating reduced neural excitability in LS^GABA^-mNAcSh^D2^-VP neurons (Figure 8, H–J). These findings complement previous reports demonstrating blunted activation of LS in rodents with depression or subjected to stress (48, 49).

To investigate the dynamic activity of LS^GABA^-mNAcSh^D2^-VP neurons associated with nerve injury–induced pain hypersensitivity and depression-like behaviors, fiber photometry was used to monitor Ca^2+^ fluctuations. First, we intracranially injected RV-mediated retrograde monosynaptic virus and Flp-dependent AAVs to express the fluorescent Ca^2+^ indicator GCaMP6s in the LS^GABA^-mNAcSh^D2^-VP neurons (Figure 8K). We observed an insignificant change in the activation of LS^GABA^-mNAcSh^D2^-VP neurons upon either von Frey or thermal stimuli in mice (Figure 8, L and M). Nevertheless, the Ca^2+^ activity of these neurons was dramatically decreased in freely moving SNI mice during the TST (Figure 8N), indicating a role of LS^GABA^-mNAcSh^D2^-VP neurons in depression-associated pathophysiology processing.

Since the population Ca^2+^ activity and the intrinsic excitability of LS^GABA^-mNAcSh^D2^-VP neurons were reduced during chronic neuropathic pain, we sought to establish a potential link between dysfunctional adaptation in LS^GABA^-mNAcSh^D2^ projection and downstream VP neurons in depression modulation. To achieve this, we first expressed functional NpHR in the LS^GABA^-mNAcSh^D2^-VP neurons in sham-operated mice (Supplemental Figure 9, A–C). Optogenetic silencing of LS^GABA^-mNAcSh^D2^-VP neurons notably produced depression-like behaviors (Supplemental Figure 9, F–H) but not pain-related behaviors (Supplemental Figure 9, D and E) in mice. Conversely, ChR2-EYFP or EYFP was expressed in the LS^GABA^-mNAcSh^D2^-VP neurons via RV-based strategies, and hM3Dq was expressed in VP glutamatergic neurons through AAVs in SNI mice (Figure 8, O and P). We found that optogenetic activation of the LS^GABA^-mNAcSh^D2^-VP neurons could not change the pain hypersensitivity in nerve-injured mice (Figure 8, Q and R). Interestingly, activation of LS^GABA^-mNAcSh^D2^-VP neurons with optogenetics robustly alleviated the depression-like behaviors (Figure 8, S–U). Notably, the antidepression-like effects were prevented by activation of VP glutamatergic neurons (Figure 8, S–U). Thus, the LS^GABA^ subpopulations contributed to processing of depression by indirectly targeting VP glutamatergic neurons.

To test whether this pathway is universal in depression modulation, we examined mice exposed to chronic restraint stress (CRS) or LPS injection, 2 rodent models of depression (Supplemental Figure 10, A and E). We observed that optogenetic activation of the LS^GABA^-mNAcSh^D2^-VP pathway could not change depression-like behaviors in either CRS mice (Supplemental Figure 10, B–D) or LPS-injected mice (Supplemental Figure 10, F–H). Together, these results show the functional causality of the LS^GABA^-mNAcSh^D2^-VP circuit in the development of depression-like phenotypes specifically in mice with chronic pain rather than in non-pain-related mouse models of depression.

## Discussion

**Figure 9:**
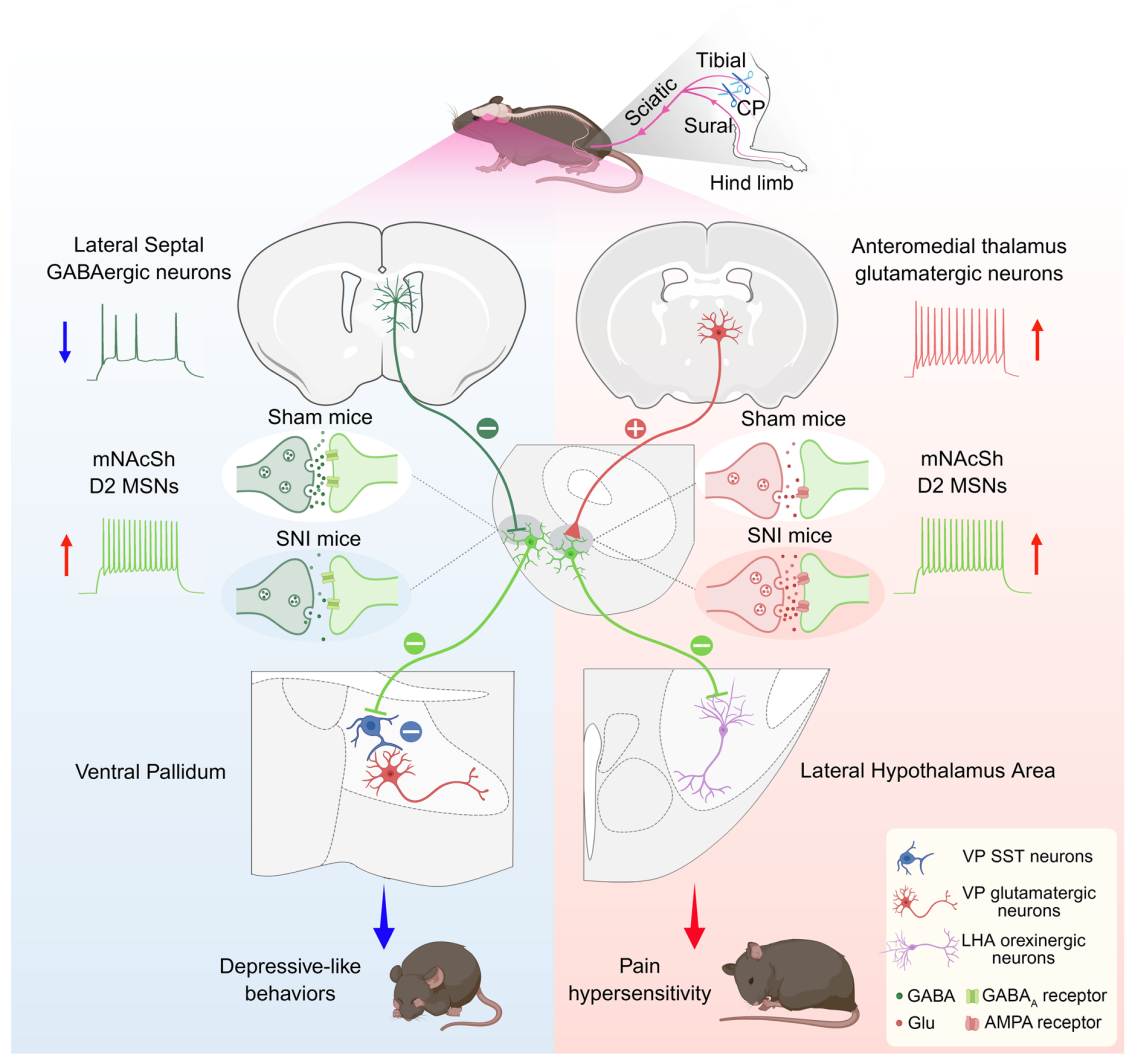
Model of distinct neural circuits that drive pain hypersensitivity and comorbid depressive-like behaviors in mice. Nerve injury produced activation of AM glutamatergic neurons projecting to the mNAcSh D2 neurons, which selectively inhibited LHA orexinergic neurons and induced pain hypersensitivity. GABAergic neurons input from the LS to D2 neurons within the mNAcSh, which disinhibit VP glutamatergic neurons by inhibiting somatostatin-expressing interneurons. Notably, this identified pathway is specifically involved in the comorbid depressive-like behaviors in mice with chronic pain. Created with BioRender.com.

Our proposed pathway model for how distinct neural circuits drive pain hypersensitivity and comorbid depression-like symptoms is shown in Figure 9. We demonstrated that neurons within the mNAcSh, more specifically the D2-expressing subpopulation, contribute to mediating pain responses and depression-like behaviors after nerve injury. These neurons received AM glutamatergic and LS GABAergic inputs and sent distinct projections to the LHA and VP, thereby modulating hypothalamic orexinergic and pallidal glutamatergic neurons, respectively. These findings suggest that the mNAcSh may function as a node for mediating pain hypersensitivity and comorbid depression-like behaviors.

Peripheral nerve injury induced chronic pain and persistent depression-like behaviors in rodents (34, 51, 52). Consistent with the previous study (34), we observed notable pain hypersensitivity and depression-like phenotypes in nerve-injured mice. Shared neural circuitry and molecular mechanisms have been implicated in the comorbidity of chronic pain and depression (53–55). For example, activation of serotonergic projections from the dorsal raphe nucleus to the central nucleus of the amygdala attenuated SNI-induced pain hypersensitivity and depression-like behaviors (34). Brain TNF drives depression-like behavior and chronic pain in arthritis (56). Interestingly, optogenetic inhibition of the BLA to anterior cingulate cortex projection selectively inhibited the chronic pain–induced depression-like behaviors, yet the pain threshold was intact (57). Consistently, the thalamic reticular nucleus to lateral habenula pathway preferably contributed to depression-like phenotypes, but not mechanical hypersensitivity, in nerve-injured mice (58). Supported by these findings, we demonstrated that divergent NAc neural circuits mediated separate behavioral features of chronic pain and comorbid depression.

Functional roles of molecularly identified subpopulations of NAc neurons have been documented (13, 30). These neurons are involved in encoding reward and aversion (14), in mediating social preference and emotion (13, 26), and in regulating pain and depression (18, 24). In the present study, the c-fos expression data and patch-clamp recording results demonstrated activation of mNAcSh neurons in SNI mice. Consistent with our findings, previous studies demonstrated activation or increased membrane excitability of the NAc in both humans and mice with chronic pain (17, 19). Interestingly, it has also been reported that chronic pain elicited decreased motivation and inhibition of the NAc D2 neurons within the NAcCore (59). The NAcLat, but not the mNAcSh, mediates stress-induced anhedonia-like behaviors (60). These studies indicated the high complexity and diversity of NAc subregions in processing different behavioral abnormalities. Indeed, anatomically distinct NAc subregions promote opposite motivational states (14).

Interestingly, although studies have demonstrated that both NAc D1 and D2 neurons responded to a variety of stimuli (61–63), we did not find notable changes in the Ca^2+^ response of these neurons in sham mice. One possible explanation is that we used subthreshold stimulus intensities (0.4 g von Frey or short radiant heat), which changed neuronal Ca^2+^ response only in SNI mice but not sham mice. Indeed, when compared with sham mice, the paw withdrawal frequencies (percentage of positive responses out of 10 stimuli) (64) to 0.4 g von Frey stimuli were substantially increased in animal models of chronic primary pain conditions (65), postoperative pain (66), neuropathic pain (67), and inflammatory pain (68). Similar to our findings, subthreshold stimulation (0.4 g von Frey) increased Ca^2+^ response of neurons in the locus coeruleus in SNI mice, but not sham controls (69). Our previous study also revealed that both subthreshold thermal and mechanical stimuli increased Ca^2+^ activity of paraventricular thalamic neurons in neuropathic pain mice (27). Supporting our speculation, we found that Ca^2+^ activity of mNAcSh neurons was increased in sham mice in response to 2.0 g von Frey, which has been demonstrated to induce notable paw withdrawal responses in naive animals (70, 71). Interestingly, the Ca^2+^ activity of mNAcSh D2 neurons was increased in SNI mice. Several previous findings were supportive of our results. For example, parabrachial nucleus (PBN) neurons were activated in response to stimulation with a 2.0 g rather than a 0.4 g von Frey filament (69). Similarly, stimuli with 1.0 g, but not 0.07 g, von Frey increased the Ca^2+^ activity of neurons within the PBN or PVT in naive mice (72–74).

We found that mNAcSh D2-MSNs specifically show increased membrane excitability in vitro and enhanced population Ca^2+^ activity in freely moving SNI mice comorbid with depression-like behaviors. Suppressing mNAcSh D2 neurons alleviates pain hypersensitivity and depression-like behaviors. These results suggest that the mNAcSh could be a node converging pain signals and affective states via D2-MSNs. It has been found that peripheral nerve injury increases the excitability of NAc shell neurons involved in the indirect pathway (putative D2-MSNs), and chemogenetic inhibition of these neurons alleviates pain behaviors (19). Additionally, the reduction of D2 receptor–mediated inhibitory tone on NAc neurons produced remarkable depression-like behaviors (23). Interestingly, although the model posits that D1- and D2-MSNs operate in opposite ways, neither our brain slice recording nor fiber photometry revealed notable changes in mNAcSh D1 neural excitability in SNI mice. The discrepancy could arise from the anatomical and functional heterogeneity of NAc MSNs (19, 30). For example, optical activation of NAc D1 neurons restored allodynia after nerve injury, yet optical inhibition of NAc D2 neurons relieved pain hypersensitivity (75). Consistent with our brain mapping data, which establishes complex neural connections between mNAcSh D2 neurons and different downstream and upstream brain regions, accumulating evidence suggests that specific input-output circuits of the NAc might underlie its different functions (14, 76). Additionally, the signals conveyed by D1- and D2-MSNs also depend on their neuronal stimulation pattern (brief vs. prolonged), psychiatric symptoms (anhedonia vs. behavioral despair), and the time course of different studies (22, 25, 76). Thus, we provide multiple lines of evidence supporting that mNAcSh D2 neurons promote behavioral abnormalities associated with pain and mood disorders.

We determined that distinct D2 projections from the mNAcSh separately mediate different aspects of symptoms related to chronic pain: the one projecting to the LHA mediates pain hypersensitivity, whereas the one projecting to the VP mediates the comorbid depression-like behaviors. The former inhibits LHA orexinergic neurons, whereas the latter disinhibits VP glutamatergic neurons. We demonstrated decreased excitability of LHA orexinergic neurons innervated by the mNAcSh D2 neurons in SNI mice, and selective manipulation of these cell populations bilaterally regulated pain thresholds. These findings support the view that the LHA acts as a brain region responsive to noxious stimuli and contributes to the modulation of pain-related behaviors (40, 41, 43).

Our electrophysiology data show that the mNAcSh D2 neurons’ projection to the VP positively modulates the excitability of VP glutamatergic neurons (Figure 5). The present anatomy data indicate that the projection rarely innervates glutamatergic neurons in the VP but synapses with a high probability onto VP SST^+^ neurons, axons of which then inhibit other VP cell types (77). Thus, it is likely that a disinhibitory circuit through the VP SST^+^ neurons can account for the positive modulation of VP glutamatergic excitability by mNAcSh D2 neurons. We further demonstrate that the excitability of VP glutamatergic neurons was increased in nerve-injured mice. Activation of these neurons reverses the antidepression-like effects induced by inhibition of mNAcSh D2 neurons in SNI-treated mice. Although disinhibition could be a simplified proposal because of the complex interactions among the heterogeneous VP cell types innervated by mNAcSh D2 terminals, these findings support the idea that mNAcSh D2 neurons contribute to the expression of depression-like behaviors at least partially through regulating VP glutamatergic neurons, which is in line with the involvement of VP glutamatergic neurons in depression-like phenotypes (46).

The distinct input patterns (Figure 6) onto the mNAcSh^D2^-LHA and mNAcSh^D2^-VP populations may partially explain the observed differential synaptic adaptations (Supplemental Figure 7) in each population. Our results do not exclude the possible contribution of other neural pathways targeting NAc D2 neurons in nerve injury–induced behavioral changes (19), yet for now it is sufficient to say that projection-specific mNAcSh D2 neuronal activity correlates with the presentation of pain hypersensitivity and comorbid depression-like symptoms.

Brain mapping results indicate that the mNAcSh^D2^-LHA projection is preferably innervated by the AM glutamatergic neurons (Figure 6). The AM is well known for its role in various functions, including sensory processing (78). Nevertheless, how the AM and its neural pathway may contribute to the processing of pain signals and depression-like behaviors remains to be elucidated. Our tracing and electrophysiological data demonstrated that AM neurons make glutamatergic synapses with mNAcSh^D2^-LHA neurons. Nerve injury, on the one hand, activates AM^Glu^-mNAcSh^D2^-LHA neurons; on the other hand, it enhances the AM–to–mNAcSh^D2^-LHA excitatory synaptic connectivity (Figure 7). Behaviorally, bidirectional manipulation of the AM^Glu^-mNAcSh^D2^-LHA pathway alleviates or mimics the SNI-induced pain, not depression-like, behaviors. Of note, chemogenetic activation of LHA orexinergic neurons abolished the pain-like phenotypes induced by optogenetic activation of AM^Glu^-mNAcSh^D2^-LHA neurons, indicating the modulatory roles of the AM^Glu^-mNAcSh^D2^-LHA^orexin^ disynaptic pathway in pain processing.

We identified that a specific population of LS GABAergic neurons preferably innervates the mNAcSh^D2^-VP neurons. This subset of LS neurons is inhibited in SNI mice comorbid with depression (Figure 8). Also, these neurons are inhibited in response to tail suspension in freely moving SNI mice. These findings are partially consistent with previous studies showing that depression-like behaviors correspond to blunted activity of LS neurons (48, 79), suggesting that LS is involved in depression-like phenotypes in mice. Indeed, optogenetic activation of the LS^GABA^-mNAcSh^D2^-VP pathway potentially relieved depression-like behaviors observed in SNI mice (Figure 8), supportive of the findings that LS neurons respond to antidepressants with increased activity (48). Interestingly, previous research suggests that chronic stress increases the number of ΔFosB-labeled neurons in LS and that activation of LS neurons causes a depression-like phenotype (80). This contradiction might be attributable to differential stress paradigms in studies. For example, single but not repeated stress increases c-fos expression in LS (81). Social trauma exclusively activates neurotensin-positive LS neurons (50), whereas chronic unpredictable stress enhances c-fos expression in GABAergic neurons, glutamatergic neurons, and cholinergic neurons within the LS (80). In support of this possible explanation, it has been shown that LS neurotensin neurons are activated (i.e., overexpressing c-fos) in susceptible mice but not in resilient mice subjected to chronic social defeat stress (CSDS) (50).

Alternatively, we hypothesized that projection-specific LS GABAergic neurons might play a unique role in regulating depression-like behavior in mice. Indeed, optogenetic activation of periaqueductal gray (PAG)-projecting neurotensin-positive LS GABAergic neurons shows no effect on social interaction in mice that underwent a subthreshold CSDS, yet chemogenetic activation of LS-PAG projection produces depression-like behaviors (50, 80). In addition, the LS^GABA^-mNAcSh^D2^-VP circuit might play relatively more important roles in chronic pain–related depressive symptoms rather than in other mouse models of depression (Supplemental Figure 10). The VP receives glutamatergic inputs from the BLA and contributes to chronic, unpredictable, mild stress–induced depression-like behaviors (82). However, VP neurons show notable heterogeneity (44, 83), with diverse neural subpopulations contributing differentially to depression regulation. Optogenetic activation of VP glutamatergic neurons projecting to the lateral habenula increases susceptibility to social defeat stress (84). Conversely, genetic ablation of glutamatergic VP neurons increases sucrose response and impairs sucrose taste aversion learning (44). Nevertheless, a specific cholinergic VP pathway mediated depression-like behaviors observed in mice with chronic pain (5). Moreover, manipulation of discrete circuits of PV neurons in the VP separately mediated either social withdrawal or behavioral despair in SDS mice, indicating that distinct VP pathways can subserve related, yet separate, behavioral abnormalities. These findings raise the possibility that the mechanisms underlying depression, for example, those related to chronic pain or stress, may be encoded by unique VP circuits.

In summary, we delineate a role of distinct mNAcSh D2 neural pathways in separately mediating pain hypersensitivity and depression-like behaviors. The present study also highlights the involvement of the projection-specific AM glutamatergic neurons and LS GABAergic neurons. How pain signals coordinate these neurons to mediate complex behavioral abnormalities is an open question for future studies. Our research provides perspectives for understanding the neural mechanisms underlying the comorbidity of pain- and depression-like phenotypes and suggests the need for more circuit-based studies in neuropsychiatric disorders to move toward symptom-based treatments.

## Methods

Detailed information on materials and methods is provided in Supplemental Methods.

### Sex as a biological variable.

Some experiments used both male and female mice. No significant differences were found between sexes. Some experiments only considered male mice. The sex and number of mice used in experiments are shown in the figure legends. Future studies will include more female mice to ensure comprehensive insights.

### Animals.

Experiments were performed on adult C57BL/6J, D1R-Cre [Tg(Drd1a-Cre) 266Gsat/Mmucd] and D2R-Cre [Tg(Drd2-cre) ER44Gsat/Mmucd] mice. Animals were obtained from GENSAT or the Laboratory Animal Center at Shanghai General Hospital. All mice were housed under a 12-hour light-dark cycle (lights on from 0700 to 1900 hours) with ad libitum access to food and water unless specified during the behavior tests. Holding and experimental room temperatures were maintained at 20°C–22°C, and humidity was maintained between 40% and 60%. All mice were allowed 1 week of acclimation to the housing facilities before the start of experiments. All mice were randomly allocated to either the control or experimental group. No difference between sexes was observed, so the data were combined. Experimenters were blind to the experimental group, and the order of testing was counterbalanced during behavioral experiments.

### Statistics.

Sample sizes in our study were not predetermined using any statistical method but were similar to those in previous publications (6, 12, 85–88). Experiments were replicated in at least 2 independent batches that yielded consistent results. The number of replicates (*n*) is indicated in the figure legends and refers to the number of experimental subjects independently treated in each experimental condition. All mice were randomly allocated to either the control or experimental group. Experiments or data analyses were blinded to treatments. All data in this study are presented as mean ± SEM. GraphPad Prism version 6 software was used for statistical analyses and graph generation. Data distribution was assumed to be normal, but this was not formally tested. Paired or unpaired 2-tailed Student’s *t* tests were carried out to compare data between 2 groups. One-way ANOVA followed by Bonferroni’s post hoc tests and 2-way ANOVA with repeated measures followed by Bonferroni’s or Tukey’s post hoc tests were used to test for statistical significance when appropriate. Statistical significance threshold was set at α = 0.05 (*P < 0.05, **P < 0.01, ***P < 0.001, and NS, *P* > 0.05).

### Study approval.

All procedures were conducted in accordance with the Guide for the *Guide for the Care and Use of Laboratory Animals* (National Academies Press, 2011) and with the approval of the IACUC at the Shanghai General Hospital of Shanghai Jiao Tong University School of Medicine.

### Data availability.

All data needed to evaluate the conclusions of this study are present in the main paper and/or in the supplement. Source data for this study are also available in the Supporting Data Values file. Any information required for analyzing the reported data is available upon request.

## Author contributions

DL, JLC, and JL contributed to the study design and wrote the paper. DL, JLC, and JL jointly supervised this work. DL, FXX, ZY, and XJH contributed to data collection and analysis. CWW and XZ conducted surgeries and histological analyses. YBZ and LJW assisted with calcium imaging experiments. All authors discussed and commented on the manuscript. The order for co–first authors was determined through discussions among the authors.

## Supplementary Material

Supplemental data

Supporting data values

## Version 1 08/01/2025

Electronic publication
