# Supplementary material for: Distinct nucleus accumbens neural pathways underlie separate behavioral features of chronic pain and comorbid depression
Source: J Clin Invest. 2025 Aug 1;135(15):e191270. doi: 10.1172/JCI191270 (PMC12321395; doi:10.1172/JCI191270)
Supplement: Supplemental data [file jci-135-191270-s014.pdf]

Supplemental Material for

Distinct nucleus accumbens neural pathways underlie separate behavioral features of chronic pain and comorbid depression

Di Liu[1,*], Fang-Xia Xu[1], Zhuang Yu[1], Xiao-Jing Huang[2], Ya-Bing Zhu[1], Li-Juan Wang[1], Chen-Wei Wu[3], Xu Zhang[1], Jun-Li Cao[4,5,*], Jinbao Li[1,*]

The file includes:

Supplemental Methods

Supplemental Figures 1 to 10

Supplemental References

Supplemental Methods

Viral constructs. For monosynaptic rabies tracing and opsin manipulation, rAAV2/9-EF1α-DIO-oRVG-WPRE-pA, rAAV2/9-EF1α-DIO-mcherry-F2A-TVA-WPRE-hGH-pA, RV-CVS-EvnA-ΔG-EGFP, and RV-CVS-EnvA-ΔG-P2A-Flp were purchased from BrainVTA. For monosynaptic antegrade tracing, rAAV2/9-EF1α-DIO-EGFP-T2A-TK-WPRE-pA and HSV-ΔTK-LSL-tdTomato were purchased from BrainVTA (Wuhan, China). For optogenetic experiments, rAAV2/9-hSyn-mCherry-WPRE-pA, rAAV2/9-EF1α-DIO-hChR2 (H134R)-mCherry-WPRE-pA, and rAAV2/9-EF1α-DIO-eNpHR3.0-mCherry-WPRE-pA were purchased from BrainVTA, AAV2/9-hEF1a-DIO-EGFP-WPRE-pA, AAV2/9-hEF1a-DIO-hChR2 (H134R)-EGFP-WPRE-pA, AAV2/9-hEF1a-fDIO-EYFP-WPRE-pA, AAV2/9-hEF1a-fDIO-hChR2 (H134R)-EYFP-WPRE-pA, and AAV2/9-hEF1a-fDIO-eNpHR3.0-EYFP-WPRE-pA were purchased from Shanghai Taitool Bioscience (Shanghai, China). For chemogenetic manipulations, rAAV2/9-hSyn-mCherry-WPRE-pA, rAAV2/9-Ef1α-DIO-hM4D (Gi)-mCherry-WPREs, and rAAV2/9-Ef1α-DIO-hM3D (Gq)-mCherry-WPREs were purchased from BrainVTA: AAV2/9-hEF1a-fDIO-mCherry-WPRE-pA, AAV2/9-hEF1a-fDIO-hM4D (Gi)-mCherry-ER2-WPRE-pA, and AAV2/9-hEF1a-fDIO-hM3D (Gq)-mCherry-ER2-WPRE-pA were purchased from Shanghai Taitool Bioscience. For fiber photometry recording, rAAV2/9-D1-CRE-WPRE-pA, rAAV2/9-D2-CRE-WPRE-pA, and rAAV2/9-EF1α-DIO-GCaMp6s-WPRE-pA were purchased from BrainVTA, and AAV2/9-CAG-fDIO-GCaMP6s-WPRE-pA was purchased from Shanghai Taitool Bioscience. For tracing soma and neural terminals, AAV2/9-hEF1a-DIO-EGFP-WPRE-pA, AAV2/2Retro-hEF1a-DIO-EYFP-WPRE-pA, and AAV2/2Retro-hEF1a-DIO-mCherry-WPRE-pA were purchased from

Shanghai Taitool Bioscience, and rAAV2/9-hSyn-DIO-mGFP-T2A-Synaptophysin-mRuby-WPRE-pA was purchased from BrainVTA. For selective ablation, rAAV2/9-EF1a-DIO-DTA-WPRE-pA was purchased from BrainVTA, and AAV2/9-hEF1a-DIO-EGFP-WPRE-pA was purchased from Shanghai Taitool Bioscience. For targeting specific cell types, rAAV-Hypocretin-CRE-WPRE-pA, rAAV-ChAT-CRE-WPRE-pA, rAAV-PV-CRE-bGH-pA, rAAV-fSST-CRE-bGH-pA, rAAV-VGLUT2-CRE-WPRE-pA, and rAAV-VGLUT2-mCherry-WPRE-pA was purchased from BrainVTA.

In vivo fiber photometry recordings. Fiber photometry was performed according to the published protocols (1-4). Briefly, after 2 weeks of virus expression, an optical fiber (200 μm OD, Hangzhou Newdoon Technology Co., Ltd) was placed in a ceramic ferrule and inserted toward the targeted regions through the craniotomy. A fiber photometry system (Thinker-Biotech) was used to record Ca^{2+} signals from neurons. An optical fiber (200 μm OD, 2 m long) guided the light between the commutator and the implanted optical fiber. The laser power was adjusted at the tip of the optical fiber to a low level of 0.01-0.02 mW to minimize bleaching (but was constant throughout each session). Generally, mice were habituated to the patch cord for 2 days. The mice were gently handled and connected to the apparatus, they were allowed to rest and habituate for 5-10 min before starting. To test the Ca^{2+} signals in response to mechanical stimuli, mice were stimulated with a 0.4-g von Frey filament in the ipsilateral hind paw. To examine Ca^{2+} signals in response to thermal stimulation, a radiant heat beam was applied to the mouse hind paw for 3 s. The Ca^{2+} fluorescence was also recorded during the tail suspension test. The calcium transient was

recorded at 50 Hz. The fluorescence values change ($\Delta F/F$) from −5 s to 10 s (0 s represents the onset of stimulation or immobility) were derived by calculating (F-F0)/F0 for each trial, where F0 was defined as the mean Ca^{2+} transients from −5 s to 0 s. MATLAB coding was used to analyze signals. The Ca^{2+} signal was heat plotted for each mouse and averaged. $\Delta F/F$ values of mice within a group were then averaged and plotted with a shaded area indicating the SEM. Histology was used to validate optical fiber implantation sites and virus expression after the experiments.

Stereotaxic injection and fiber implantation. Mice were anesthetized by vaporized sevoflurane (induction, 2.5%; maintenance, 1.5%) and head-fixed in a mouse stereotaxic apparatus (RWD Life Science Co.). Mouse eyes were lubricated using ophthalmic ointment throughout the surgery. A small cut was made on the skin at the craniotomy location, and the muscles were removed. Around 0.25-mm^2 craniotomy window was made for each region using a dental drill. ~100 nl of the viral solution was infused through a glass pipette (pulled using Model P-97, Sutter Instrument; tip diameter, 10-20 μm) at a rate of 20-25 nL/min. For the AAV injections, viruses were bilaterally or unilaterally injected into the LS (AP + 0.50 mm, ML + 0.40 mm, DV −3.50 mm), AM (AP - 0.70 mm, ML + 0.75 mm, DV −3.75 mm), mNAcSh (AP + 1.58 mm, ML + 0.45 mm, DV −4.80 mm), LH (AP – 1.50 mm, ML + 1.1 mm, DV −4.90 mm), and VP (AP + 0.20 mm, ML + 1.45 mm, DV −5.00 mm). After each injection, the cannula remained at the injection site for 5 min, after which it was slowly removed to avoid viral backflow. One week before the behavioral tests, fiber implantation was performed. For fiber photometry recordings, optical fibers (200 μm OD, Hangzhou

Newdoon Technology Co., Ltd) were unilaterally implanted. For the optogenetic manipulations, optical fibers (200 μm OD, Hangzhou Newdoon Technology Co., Ltd) were bilaterally implanted (200-300 μm above the viral injection coordinates). After implantation, all fibers were made to adhere to the skull with dental cement. Mice were allowed to recover for at least 1 week. Typically, tracing, fiber photometry, electrophysiological, or behavioral experiments were performed 3 weeks later.

Electrophysiological Recordings.

Acute brain slice preparation. The electrophysiological experiments were performed using methods previously described (5-7). Mice were anesthetized with sevoflurane and perfused with the ice-cold sucrose-based solution. After decapitation, brains were transferred quickly into a sucrose-based cutting solution bubbled with 95% O_2 and 5% CO_2 and maintained at ~4 °C. The sucrose-based cutting solution contained the following: 254 sucrose, 3 KCl, 1.25 NaH_2PO_4, 24 $NaHCO_3$, 10 D-glucose, 2 $CaCl_2$, and 2 $MgCl_2$, aerated with 95/5% O_2/CO_2. Coronal brain slices (300 μm) were prepared using a Leica VT1200S vibratome (Leica Biosystems) in the cutting solution. Slices were maintained in holding chambers filled with aCSF containing the following (in mM): 128 NaCl, 3 KCl, 1.25 NaH_2PO_4, 24 $NaHCO_3$, 10 D-glucose, 2 $CaCl_2$, and 2 $MgCl_2$, equilibrated with 95/5% O_2/CO_2. The aCSF pH was maintained within 7.3-7.4, and osmolarity was adjusted to 300-305 mOsm. The slices were allowed to recover at 32-34°C for 1 h and then maintained at room temperature. All solutions were continuously boiled with 95% O_2 and 5% CO_2 throughout slice preparation and electrophysiological recordings.

Ex vivo electrophysiology. For path-clamp recordings, brain slices were placed in a recording chamber for electrophysiological recording with continuous perfusion of oxygenated standard aCSF at 2.5-3 ml min^{-1}. Patch pipettes (4-7 MΩ) were pulled from thin-walled borosilicate glass (World Precision Instruments, Inc.) using a micropipette puller (P-97, Sutter Instruments). Neurons were visualized using an upright microscope with an IR-DIC lens and illuminated with a white light or LED. For whole-cell recordings, patch pipettes were filled with an internal solution containing the following (in mM): 115 potassium gluconate, 10 mM HEPES, 10 mM phosphocreatine, 1.5 mM $MgCl_2$, 20 mM KCl, 2 mM Mg-ATP, and 0.5 mM GTP (pH 7.2, 285 mOsm). To measure the intrinsic membrane properties of neurons, the spikes were induced by incremental current injection (each step increase was 10 or 20 pA). For ChR2 experiments, sustained and trains (1-20 Hz) of blue light (473 nm) were delivered to neurons expressing ChR2. For NpHR experiments, yellow light (589 nm) was delivered to neurons expressing NpHR. To measure the function of chemogenetic viruses, neurons expressing hM3Dq-mCherry or hM4Di-mCherry were recorded. For chemogenetic activation, neurons were recorded in the current clamp model. For chemogenetic inhibition, mCherry-labeled neurons were injected with depolarization currents, and the number of activated action potentials was calculated before and 3 min after bath application of CNO.

Evoked postsynaptic currents were elicited by 2-ms blue light stimulation of axon terminals of neurons infected with ChR2. Blue-light-evoked postsynaptic currents were recorded with Cs^+-based peptide solution (in mM: 140 CsMeSO4, 20 HEPES, 0.4 Cs-EGTA, 2.5 Mg-ATP, 0.5 GTP, and 1 QX-314, 290 mOsm, adjusted to 7.4 with CsOH).

EPSCs and IPSCs were recorded when the membrane potential was held at -70 mV and 0 mV, respectively. To test whether the recorded EPSCs were mediated by AMPA/kainite receptors, 10 μM NBQX was added to the aCSF. To test whether the recorded IPSCs were mediated by $GABA_A$ receptors, 50 μM Bicuculline was added to the aCSF. To test whether the optic-evoked postsynaptic currents were elicited by direct synaptic connections, 0.5 μM tetrodotoxin (TTX) and 100 μM 4-aminopyridine (4-AP) were added to the aCSF. For evaluating the presynaptic mechanism, paired pulses with an interval of 50 or 100 ms were delivered, and the PPR was calculated as the amplitude ratio $EPSC_2/EPSC_1$. To measure the AMPA/NMDA current ratio, neurons were first clamped at −70 mV, and the AMPA receptor-mediated EPSCs were recorded. To record the NMDA receptor-mediated EPSCs, neurons were clamped at +40 mV in the presence of bicuculline, and EPSCs were determined 50 ms after the onset of the evoked current (8).

Voltage-clamp and current-clamp recordings were carried out using a Multiclamp 700B amplifier (Molecular Devices, USA). Traces were low-pass filtered at 2 kHz and digitized at 10 kHz. For light stimulation, light pulses were delivered through digital commands from the Digidata 1550B (Molecular Devices, USA) and the Digital Stimulator. Data acquisition and analysis were performed using Clampfit 10.6 software (Molecular Devices, USA). All recordings were performed blind to experimental conditions.

Optogenetic manipulation. Mice were handled daily for 2 days before the behavioral tasks. On the day of the experiment, mice were transported to a testing room and habituated for approximately 1 h. For blue (ChR2) and orange (eNpHR3.0) light stimulation,

the chronically implanted optical fibers (diameter 200 μm, Hangzhou Newdoon Technology Co., Ltd) were connected to either a 473 nm blue laser diode or a 589 nm orange laser diode using a patch cord with an FC/PC adaptor, after which mice were returned to the home cage for at least 30 min. Next, either blue light (20 Hz, 473 nm, 3-5 mW, 5-ms pulses) or yellow light (589 nm, 3-5 mW, constant) was delivered to activate or inhibit the soma and circuits under selective control of an intelligent optogenetic system.

DREADD manipulation. In the experiments using the designer receptor exclusively activated by designer drugs (DREADD), Clozapine-N-oxide (CNO, MedChemExpress) was dissolved in dimethyl sulfoxide (DMSO) and then diluted with 0.9% saline to a final concentration of 0.001% DMSO. Animals expressing hM4D (Gi) or hM3D (Gq) received an intraperitoneal (i,p.) injection of CNO (1.0 mg kg^{-1}) 20 min before the behavior tests.

Mouse model of neuropathic pain. According to previously described methods (9), after baseline pain threshold tests, the SNI surgery was performed under anesthesia with sevoflurane in mice. The skin and muscle of the left thigh were incised to explore the sciatic nerve, consisting of the sural, common peroneal, and tibial nerves. After exploration, the tibial nerves and common peroneal were tightly ligated with a 6/0 silk suture (Ethicon) and transected together. Then the nerves were transected, and about 1 mm sections from the dot were removed. After surgery, the overlaying muscles and skin were closed with 6/0 and 4/0 silk sutures. In sham-operated mice, the procedure was the same as for the SNI group, but without ligation and transection of the tibial nerves and common peroneal. 10-14 days

after SNI surgery, SNI mice displaying reduced mechanical threshold and thermal withdrawal latency were included in further experiments.

Mouse model of inflammatory pain. To establish a mouse model of inflammatory pain (10, 11), under anesthesia with sevoflurane, the mouse received an intra-plantar injection of Complete Freund's adjuvant (CFA) (10 μl, F5881, Sigma-Aldrich) using an insulin syringe. Sham groups received an intra-plantar injection of Saline (0.9% NaCl, 10 μl).

Animal models of depression

Chronic restraint stress (CRS). To establish a CRS mouse model of depression (12), mice were constrained from moving by placing them in a 50-ml conical tube for 3 hours every day for 3 weeks. Holes were drilled in the ends of the tube for mouse breathing. To exclude the effect of acute stress, the mice were allowed to rest for 1 day after CRS before the behavioral experiments. The control mice were allowed to move freely in the cage without water or food during the restraint period of CRS mice.

LPS-induced depression. The LPS-induced depression model was improved and used according to the previous description (12, 13). LPS (Sigma, L-2880) was dissolved in sterile 0.9% saline and was intraperitoneally (i.p.) injected into mice at a dosage of 0.5 mg/kg. Sham group mice were intraperitoneally injected with saline. Saline or LPS was injected daily for 7 days. Behavioral tests were conducted 24 hours after the last injection.

RNAscope in situ hybridization. Mice were anesthetized with sevoflurane and

transcranial perfused with saline, followed by 4% PFA. Brain tissue was extracted and post-fixed with 4% PFA for 1 hour and then immersed in 30% sucrose solution at 4 °C overnight. Mouse brain samples were embedded in an OCT compound (Tissue-Tek). Coronal slices (20 μm thickness) were prepared on a cryostat (Leica). Fluorescence in situ hybridization was performed using an RNAscope Multiplex Fluorescent Reagent Kit and appropriately designed probes (ACD Bio). In brief, according to the manufacturer's standard protocol, brain slices mounted on slides were treated with a series of dehydration steps using 50%, 70%, and 100% ethanol. Tissue sections were permeabilized with ACD protease IV for 30 min, with slides then hybridized with a prewarmed probe in the HybEZ oven at 40 °C for 2 h. The mouse ACD probes used included Mm-Drd1 (cat. no. 461901) and Mm-Drd2 (cat. no. 406501). After hybridization, brain sections went through multiple steps of signal amplification and fluorescence labeling. Sections were stained with DAPI and stored at 4 °C. Stained sections were imaged with a 20× magnification objective on a Leica microscope.

Behavioral tests. The following behavioral experiments were performed in a blinded manner. The room temperature and humidity remained stable for all experiments. The mice were habituated to the testing room for at least 60 min on each test day.

von Frey test. The von Frey test was used to determine the mechanical sensory threshold (14). Mice were confined in boxes placed on an elevated metal mesh floor, and a sequence of von Frey hairs (0.16-2.00 g, Stoelting, Wood Dale, IL) was employed to perpendicularly stimulate the mouse hind paw with logarithmically increasing stiffness. The 50% paw

withdrawal threshold (PWT) was calculated, conforming to the up-down method.

Hargreaves test. The paw withdrawal latency (PWL) test was used to measure thermal nociception as described previously (15, 16). Mice were placed in an individual plexiglass box with a glass floor. A radiant heat beam was exposed directly to the hind paw until the paw was withdrawn. The test was executed with a 20-second cutoff time to avoid potential damage. The trials were in triplicate for each mouse at an interval of 5 min.

Open field test. The open field test (OFT) is employed to assess the spontaneous locomotor activities of mice (3). An open-topped white box (40 × 40 × 60 cm) was placed in a sound-attenuated box. A tracking system (Ningbo AnLai, China) was used to analyze animal behaviors. At the start of the test, each animal was placed in the central area. The movement of the animal was recorded with a camera attached to a computer, and the mouse's body center was tracked for 6 min. The distance traveled and the total velocity of the mouse were calculated. The box was cleaned with 70% ethanol after each trial.

Sucrose preference test. To examine depressive-like phenotypes, the sucrose preference test (SPT) was conducted as previously described (3). In brief, after adapting to a single cage, mice were water-deprived for 24 hours and then exposed to one bottle of normal drinking water and one bottle of 1% sucrose solution for 2 hours. Bottle positions were switched after 1 hour. The total consumption of sucrose and drinking water was measured at the end of the session by weighing the bottles. Sucrose preference was defined as the ratio of the consumption of sucrose versus the consumption of both normal drinking water and sucrose solution.

Tail suspension test. The tail suspension test (TST) is a commonly employed behavioral

assay to assess despair-like behaviors in mice (8). Animals were suspended by the tail on a horizontal bar (60 cm above the floor) for 5 minutes using adhesive tape placed roughly 1.5 cm from the tip of the tail. Mice were considered immobile when there were no initiated movements. The cumulative immobility time was recorded by an investigator unknown of the treatments.

Forced swimming test. The Forced Swimming Test (FST) is a behavioral assessment tool used to evaluate despair-like behaviors in mice (3). This test was performed in a clear glass cylinder (height 30 cm, diameter 19 cm) filled with fresh water at ~25℃. Animals were gently and individually released into the water. The water depth (15 cm) was set to prevent mice from touching the bottom with their hind limbs or tails. Immobility time was measured for 5 min by blind manual scoring. Despair behavior was defined as ceasing struggling and remaining floating in the water, making only movements necessary for keeping its nose above the water. After testing, mice were placed in a pre-warmed cage for 30 min before they were returned to their home cages.

Immunohistochemical staining. For the c-Fos assay, the mice were perfused 90 min after paw withdrawal threshold tests. Mice were deeply anesthetized with sevoflurane and then sequentially perfused with saline and 4% paraformaldehyde solution. Brains were dissociated and post-fixed in 4% paraformaldehyde solution at 4 °C overnight and then immersed in 20% and 30% sucrose solution at 4 °C for 24 hours for dehydration. Frozen brains were cut into 40 mm coronal slices with a cryostat microtome system (Leica) at -20 °C. Sections were stored at 4 °C in PBS until they were processed. For staining, the

brain slices were washed 3 times with PBS (5 min each) and blocked with 10% normal donkey serum at room temperature for 1 hour in PBS with 0.3% Triton X-100. The primary antibody incubation was performed by incubating the sections overnight at 4 °C in a PBS solution containing 0.3% Triton X-100 and donkey serum, polyclonal anti-c-fos (1:1000, rabbit, Abcam, cat. no. ab190289, RRID: AB_2737414), anti-orexin (1:500, Santa-Cruz, cat. no. sc-80263, RRID: AB_1126868), anti-Glutamate (1:300, rabbit, Sigma-Aldrich, cat. no. G6642, RRID: AB_259946), and anti-GABA (1:300, rabbit, Sigma-Aldrich, cat. no. A2052, RRID: AB_477652). Next, sections were washed 3 times with PBS (10 min each). The secondary antibody incubation was performed for 2 hours at room temperature protected from light using Alexa Fluor 488 donkey anti-mouse IgG (1:500, Jackson ImmunoResearch, cat. no. 715545150; RRID: AB_2340846), Alexa Fluor 488 donkey anti-rabbit IgG (1:500, Jackson ImmunoResearch, cat. no. 711545152; RRID: AB_2313584), and Alexa Fluor 647 donkey anti-rabbit IgG (1:500, Jackson ImmunoResearch, cat. no. 711605152; RRID: AB_2492288). Finally, sections were mounted with an antifade mounting medium containing 4',6-diamidino-2-phenylindole (DAPI) (Beyotime Biotechnology, cat. no. P0131), which was used to stain Nuclei and was shown in the blue channel. Tissue sections were imaged with a 10× or 20×magnification objective on a Leica microscope. Quantitative data, such as analysis of cell counts and colocalization, were conducted by an observer blind to the condition.

Supplemental Figures and Legends

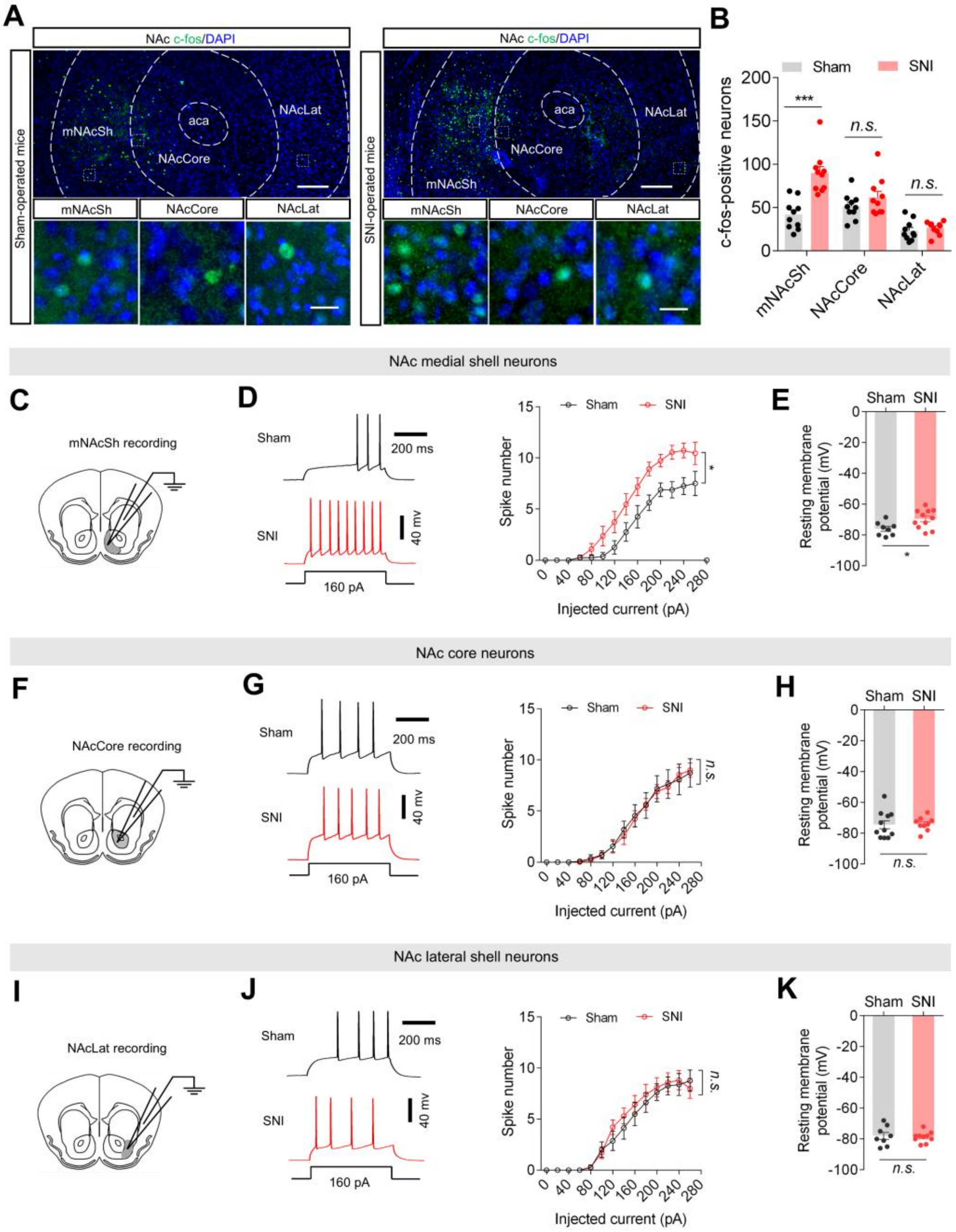

Supplemental Figure 1. Enhanced neural excitability in the mNAcSh of SNI 6-weeks mice. (**A**) Mice were perfused 90 min after paw withdrawal threshold tests. Fluorescence images showing expression of c-Fos (green) in sham (left) and SNI mice (right). Scale bar: top, 200 μm; bottom, 20 μm. (**B**) Statistical analysis of the c-Fos expressing neurons in sham and SNI mice (n = 3 male and 2 female mice/group). Two-way repeated-measures ANOVA test with multiple comparisons, ***p < 0.001, not significant (*n.s.*). (**C**) Whole-cell patch-clamp recording from mNAcSh neurons in brain slices. (**D**) Example traces (left) and average input-output function (right) of membrane potential responses to step current injections in mNAcSh neurons from sham-operated (black) and SNI-treated (red) mice (n = 8 and 11 cells from 2 male and 2 female animals/group). Two-way repeated-measures ANOVA, column factor $F_{(1, 17)}$ = 8.107, *p = 0.0111. (**E**) The resting membrane potential (RMP) of mNAcSh neurons in sham mice (black) and SNI mice (red) (n = 8 and 11 cells

from 2 male and 2 female animals/group). Two-tailed *t*-test, t_{17} = 2.539, *p = 0.0212. (**F**) Whole-cell recording from NAcCore neurons in brain slices. (**G**) Example traces (left) and summarized data (right) of membrane potential responses to step current injections in NAcCore neurons from sham mice (black) and SNI mice (red) (n = 11 and 9 cells from 2 male and 2 female animals/group). Two-way repeated-measures ANOVA, interaction $F_{(13, 234)}$ = 0.0998, p > 0.9999. (**H**) The RMP of NAcCore neurons in sham (black) and SNI (red) mice (n = 11 and 9 cells from 2 male and 2 female animals/group). Two-tailed *t*-test, t_{18} = 0.1776, p = 0.8610. (**I**) Whole-cell recording from NAc lateral shell neurons in brain slices. (**J**) Representative traces (left) and summarized data (right) of membrane potential responses to step current injections in NAcLat neurons from sham (black) and SNI (red) mice (n = 8 and 10 cells from 2 male and 2 female animals/group). Two-way repeated-measures ANOVA, interaction $F_{(13, 208)}$ = 0.6533, p = 0.8065. (**K**) The RMP of NAcLat neurons in sham (black) and SNI (red) mice (n = 8 and 10 cells from 2 male and 2 female animals/group). Two-tailed *t*-test, t_{16} = 0.3407, p = 0.7378. Data are represented as mean ± SEM.

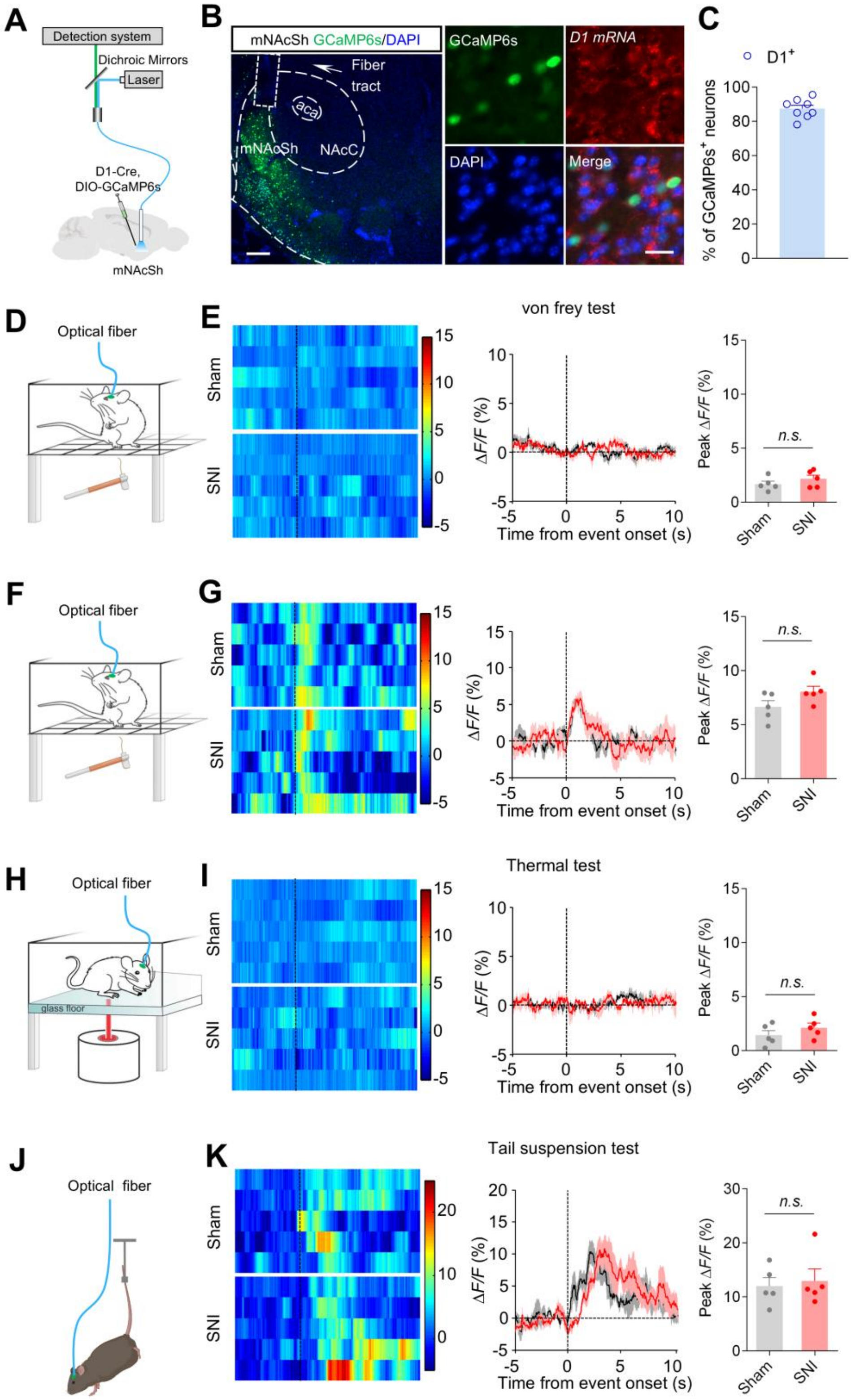

Supplemental Figure 2. Insignificant changes in the Ca^{2+} response of mNAcSh D1 neurons to pain- and depression-related stimuli. (**A**) Scheme showing viral injection to express GCaMP6s in mNAcSh of D1-Cre mice for fiber photometry. (**B** and **C**) Representative images (**B**) and quantitation (**C**) of *D1 mRNA* expression in GCaMP6s-positive mNAcSh neurons. Scale bar: left, 200 μm; right, 25 μm. (**D**) Scheme of the fiber photometry apparatus used to measure 0.4-g von Frey fiber-evoked Ca^{2+} signals in

mNAcSh D1 neurons. (**E**) Left: heat maps showing the Ca^{2+} signals evoked by 0.4g von Frey fiber in mNAcSh D1 neurons from sham-treated and SNI-operated mice. Middle: averaged responses of mice in different groups. Right: peak $\Delta F/F$ of Ca^{2+} signals in different groups (n = 3 male and 2 female animals/group). Two-tailed *t*-test, p = 0.3048. (**F**) Scheme of the fiber photometry in mice during stimulation with a 2.0-g von Frey fiber. (**G**) Heat maps (Left), average calcium activity (Middle), and quantification of peak ΔF/F (Right) of Ca^{2+} signals in NAc D1 neurons from mice upon stimulation with a 2.0-g von Frey filament (n = 5 mice /group). Two-tailed t-test, p = 0.1021. (**H**) Scheme of the fiber photometry apparatus used to measure thermal pain-evoked Ca^{2+} signals in mNAcSh D1 neurons. (**I**) Left: heat maps (left) showing the Ca^{2+} signals evoked by thermal stimulation in mNAcSh D1 neurons from sham-treated and SNI-operated mice. Middle: averaged responses of mice in different groups. Right: peak $\Delta F/F$ of Ca^{2+} signals in different groups (n = 3 male and 2 female animals/group). Two-tailed *t*-test, p = 0.2805. (**J**) Scheme of the fiber photometry apparatus used to measure depression-related, stimulation-evoked Ca^{2+} signals in mNAcSh D1 neurons. (**K**) Left: heat maps (left) showing the Ca^{2+} signals during the tail suspension test in mNAcSh D1 neurons from sham-treated and SNI-operated mice. Middle: averaged responses of mice in different groups. Right: peak $\Delta F/F$ of Ca^{2+} signals in different groups (n = 3 male and 2 female animals/group). Two-tailed *t*-test, p = 0.7289. Data are represented as mean ± SEM.

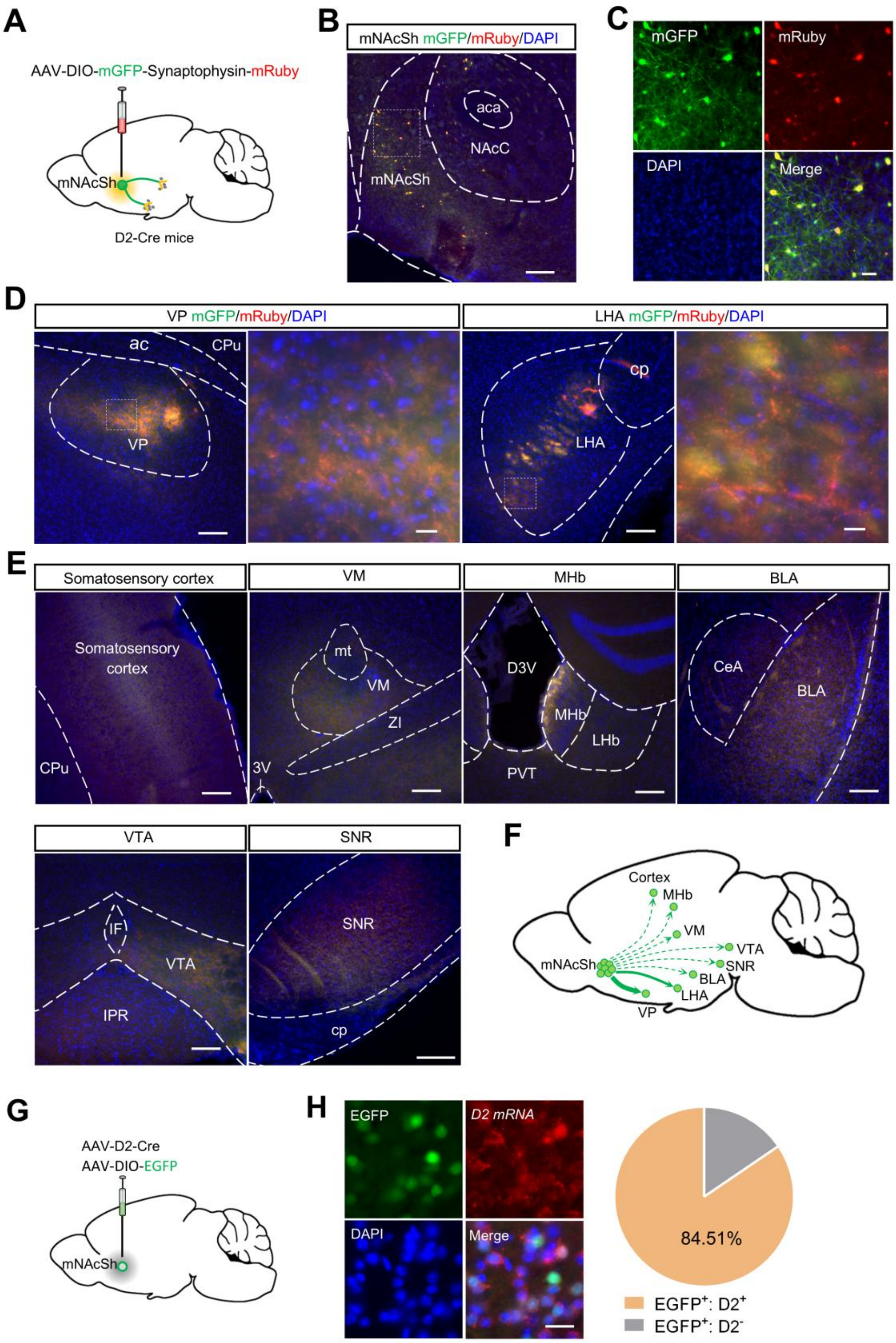

Supplemental Figure 3. Brain mapping the downstream targets of the mNAcSh D2 neurons. (**A**) Schematic of strategy for tracing the targets of mNAcSh D2 neurons. (**B** and C) Representative image (**B**) and magnification (**C**) of the injection site in mNAcSh. Scale bar, 200 μm; inset, 25 μm. (**D** and **E**) Fibers and synaptic puncta in VP and LHA (**D**), somatosensory cortex, ventromedial thalamic nucleus (VM), medial habenular nucleus

(MHb), anterior part of basolateral amygdaloid nucleus (BLA), ventral tegmental area (VTA), and substantia nigra (SNR) (**E**) from mNAcSh D2-MSNs. Scale, 200 µm; inset, 20 µm. (**F**) Schematic showing summary of the outputs of mNAcSh D2 neurons. (**G**) Schematic showing viral injection. (**H**) Representative images and the plot reveal co-expression of *D2 mRNA* (red) in EGFP-positive neurons labeled in a Cre-dependent manner. Scale, 20 µm.

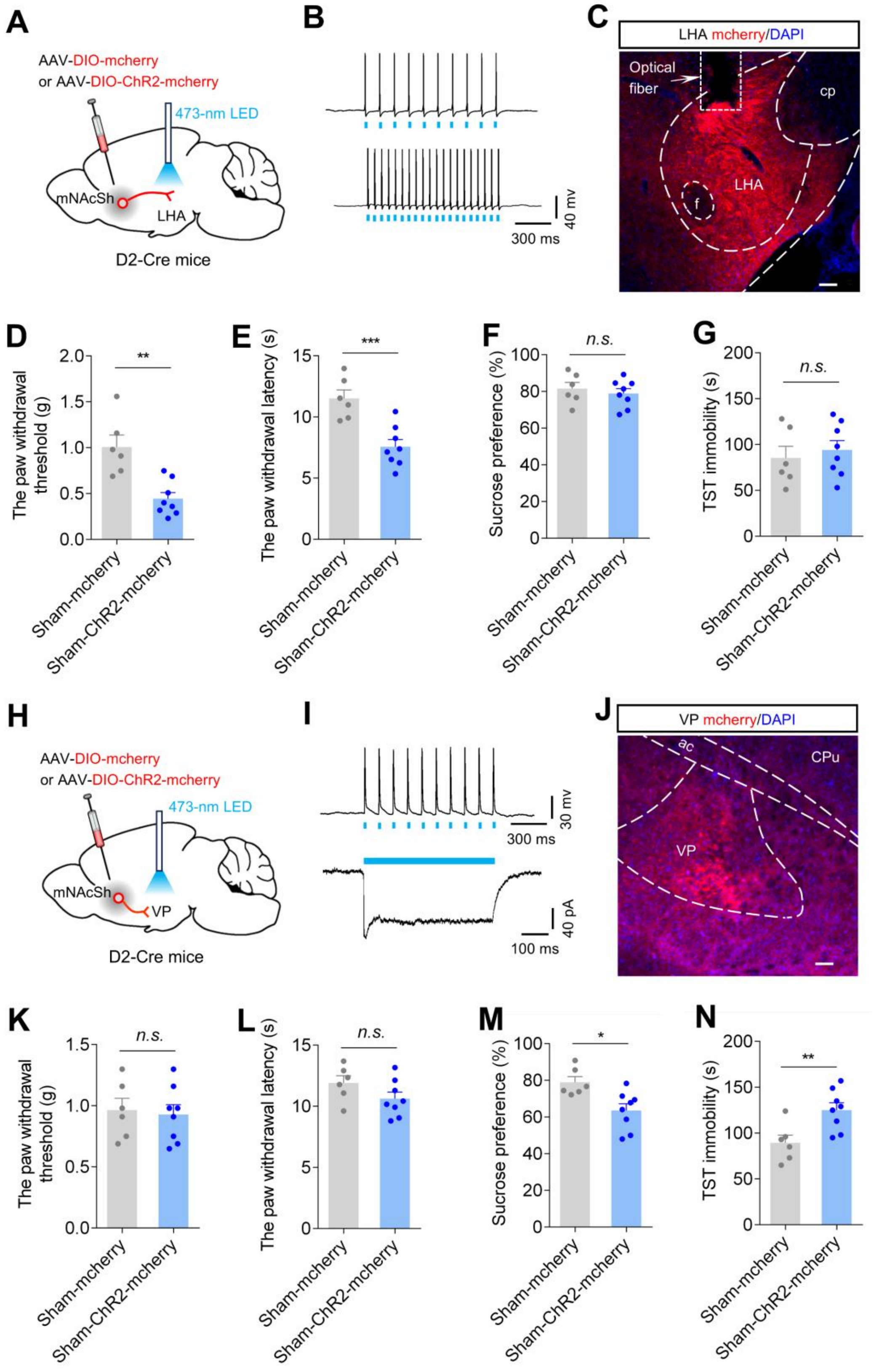

Supplemental Figure 4. Activation of mNAcSh^{D2}-LHA and mNAcSh^{D2}-VP projections induces pain-like and depressive-like phenotypes in sham-operated mice. (**A**) Schematic showing viral injection. (**B**) Example traces of spiking of a recorded mNAcSh^{D2}-LHA neuron to pulses of blue light at 10 and 20 Hz. (**C**) A representative image showing

ChR2-mcherry-expressing mNAcSh D2 terminals in LHA and the optical fiber track. Scale bar: 100 µm. (**D** and **E**) The mechanical paw withdrawal threshold (**D**) and thermal paw withdrawal latency (**E**) in mcherry control and ChR2-expressing sham-operated animals (n = 6-8 mice per group). Two-tailed *t*-test; t_{12} = 4.159, ***p* = 0.0013 (**D**); t_{12} = 4.382, ****p* = 0.0009 (**E**). (**F** and **G**) Percentage of sucrose water consumption in SPT (**F**) and immobility time in TST (**G**) in mCherry control and ChR2-expressing mice (n = 6-8 mice per group). Two-tailed *t*-test; t_{12} = 0.6318, *p* = 0.5394 (**F**); t_{12} = 0.5472, *p* = 0.5943 (**G**). (**H**) Schematic showing viral injection. (**I**) Example traces of spiking (top) in response to blue light at 10 Hz and inward current (bottom) induced by 500 ms blue light illumination in a recorded mNAcSh^{D2}-VP neuron. (**J**) Example image showing ChR2-mcherry-labeled mNAcSh D2 axonal terminals in the VP. Scale bar: 100 µm. (**K** and L) Mechanical paw withdrawal threshold (**K**) and thermal paw withdrawal latency (**L**) in mCherry control and ChR2-expressing mice (n = 6-8 mice per group). Two-tailed *t*-test; t_{12} = 0.3009, *p* = 0.7686 (**K**); t_{12} = 1.614, *p* = 0.1326 (**L**). (**M** and **N**) Percentage of sucrose water consumption in SPT (**M**) and immobility time in TST (**N**) in mice (n = 6-8 mice per group). Two-tailed *t*-test; t_{12} = 3.039, **p* = 0.0103 (**M**); t_{12} = 3.061, ***p* = 0.0099 (**N**). Data are represented as mean ± SEM.

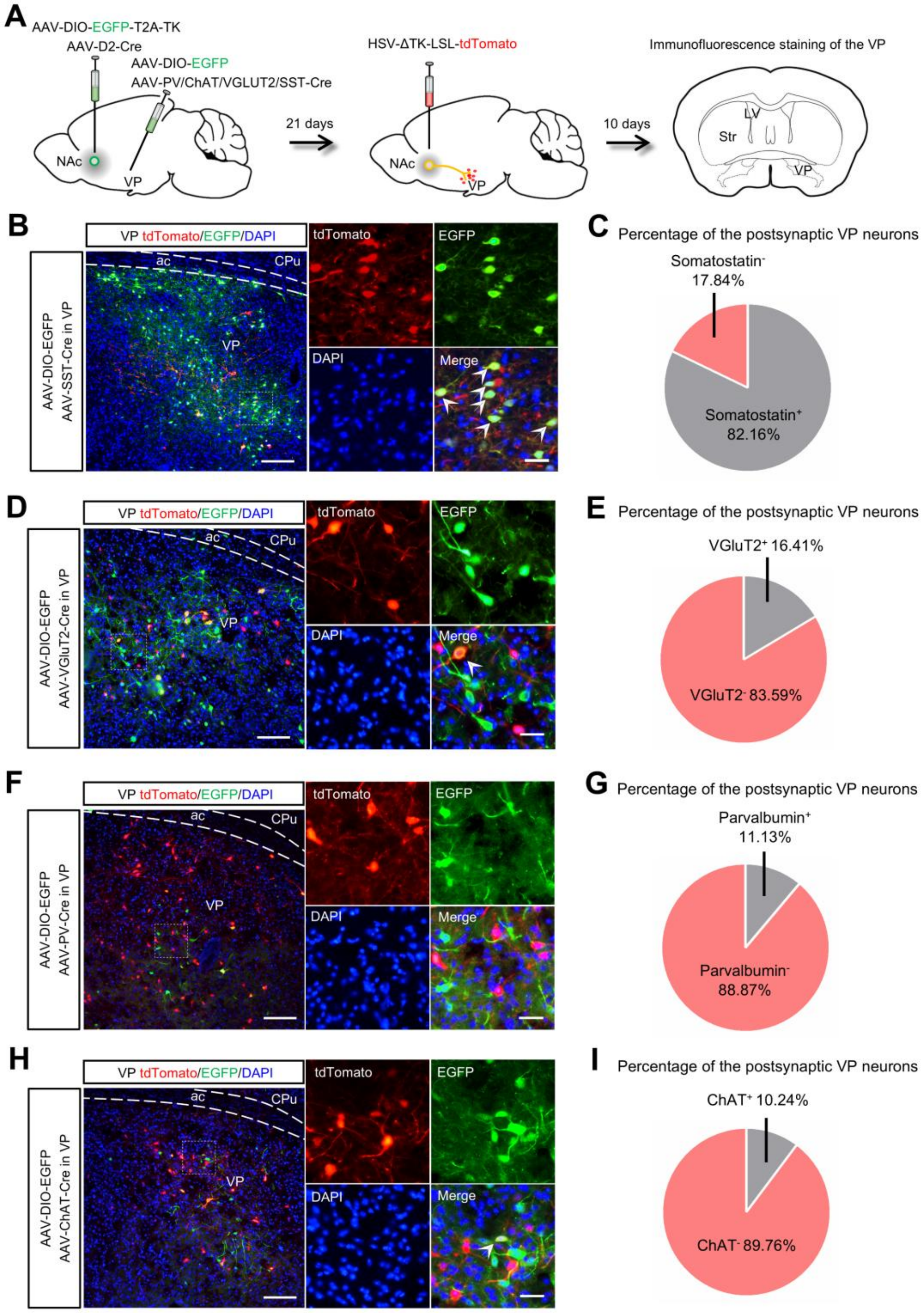

Supplemental Figure 5. Mapping the downstream VP neural subpopulations of mNAcSh D2 neurons. (**A**) HSV-based labeling of the postsynaptic VP neurons and genetically defined neuron types in the VP. (**B**) Fluorescence images showing HSV-labeled postsynaptic neurons (tdTomato) and EGFP-expression SST^{+} neurons (EGFP) in the VP area. (**C**) Quantification of the percentage of SST-positive and negative neurons in VP HSV-tdTomato cells. (**D**) Fluorescence images showing HSV-labeled postsynaptic neurons (tdTomato) and EGFP-expressing VGLUT2^{+} neurons (EGFP) in the VP area. (**E**) Quantification of the percentage of VGLUT2-positive and negative neurons in VP HSV-

tdTomato cells. (**F**) Fluorescence images showing HSV-labeled postsynaptic neurons (tdTomato) and EGFP-expressing PV^+ neurons (EGFP) in the VP area. (**G**) Quantification of the percentage of Parvalbumin-positive and negative neurons in VP HSV-tdTomato cells. (**H**) Fluorescence images showing HSV-labeled postsynaptic neurons (tdTomato) and EGFP-expressing $ChAT^+$ neurons (EGFP) in the VP area. Scale, 100 and 20 μm. (**I**) Quantification of the percentage of ChAT-positive and negative neurons in VP HSV-tdTomato cells.

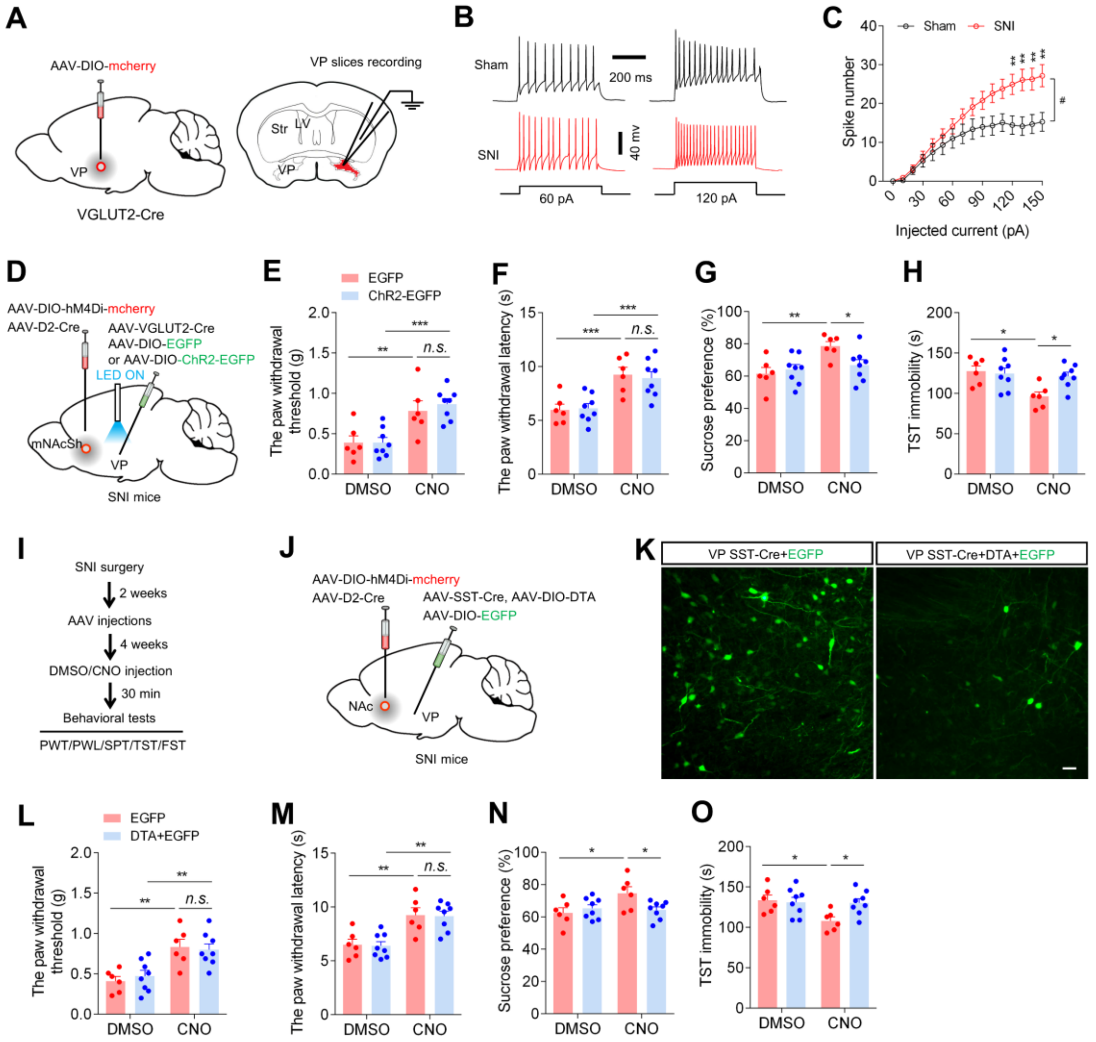

Supplemental Figure 6. The antidepressive-like effects induced by chemogenetic silencing of mNAcSh D2 neurons were reversed by optogenetic activation of VP VGLUT2 neurons or ablation of VP SST^{+} neurons. (**A**) Illustration revealing labeling of VP VGLUT2 neurons for patch-clamp recording. (**B**) Example traces of membrane potential responses to step current injections in a sham (black) and SNI (red) glutamatergic neuron. (**C**) Average input-output function of glutamatergic neurons in sham control (black) and SNI-treated (red) animals (n = 13 and 14 cells, from four animals per group). Two-way RM ANOVA with Bonferroni's multiple comparisons test; $F_{(15, 375)} = 7.987$, $p < 0.0001$; *$p < 0.05$ and **$p < 0.01$. (**D**) Schematic showing viral injection to express hM4Di in mNAcSh D2 neurons and express EGFP-control or ChR2-EGFP in VP glutamatergic neurons, respectively. (**E** and **F**) Mechanical paw withdrawal threshold (**E**) and thermal paw withdrawal latency (**F**) in SNI mice with (CNO) or without (DMSO) chemogenetic inhibition of mNAcSh D2 neurons (n = 6-8 mice per group). Two-way repeated-measures ANOVA with Bonferroni's multiple comparisons test; time factor $F_{(1, 12)} = 34.56$, $p < 0.0001$ (**E**); time factor $F_{(1, 12)} = 81.80$, $p < 0.0001$ (**F**); **$p < 0.01$, ***$p < 0.001$. (**G** and **H**) Percentage of sucrose water consumption in SPT (**G**) and immobility time in TST (**H**) (n = 6-8 mice per group). Two-way repeated-measures ANOVA with Bonferroni's multiple comparisons test; interaction $F_{(1, 12)} = 7.919$, $p = 0.0156$ (**G**); group factor $F_{(1, 12)} = 7.282$, $p = 0.0194$ (**H**); *$p <$

0.05, **$p < 0.01$. (**I**) Time course for experimental design. (**J**) Schematic diagram for DTA-mediated ablation of VP SST^+ neurons and AAV-based expression of hM4Di in mNAcSh D2 neurons. (**K**) Images for EGFP-labeled neurons in VP with (right) or without DTA (left) injection. Scale bar, 20 μm. (**L** and **M**) Effects of ablating VP SST^+ neurons on the mechanical paw withdrawal threshold (**L**) and thermal paw withdrawal latency (**M**) in SNI mice with (CNO) or without (DMSO) chemogenetic inhibition of mNAcSh D2 neurons (n = 6-8 mice per group). Two-way repeated-measures ANOVA with Bonferroni's multiple comparisons test; time factor $F_{(1, 12)} = 28.94$, $p = 0.0002$ (**L**); time factor $F_{(1, 12)} = 33.43$, $p < 0.0001$ (**M**); **$p < 0.01$. (**N** and **O**) Percentage of sucrose water consumption in SPT (**F**) and immobility time in TST (**N**) (n = 6-8 mice per group). Two-way repeated-measures ANOVA with Bonferroni's multiple comparisons test; interaction $F_{(1, 12)} = 7.286$, $p = 0.0193$ (**F**); interaction $F_{(1, 12)} = 5.363$, $p = 0.0391$ (**O**); *$p < 0.05$. Data are represented as mean ± SEM.

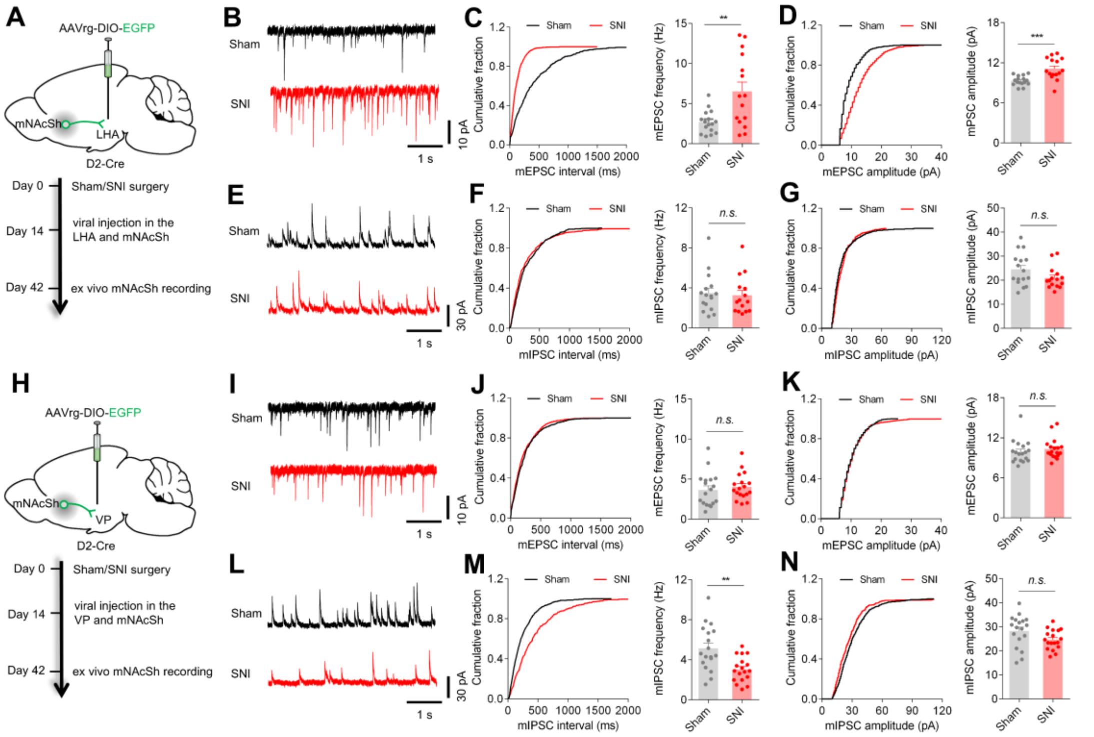

Supplemental Figure 7. Differential changes in synaptic activity in mNAcSh^{D2}-LHA and mNAcSh^{D2}-VP neurons in SNI mice. (**A**) Schematic diagram for projection-specific labeling of mNAcSh^{D2}-LHA neurons (top) and the time course for experimental design (bottom). (**B**) Representative traces of mEPSC in mNAcSh^{D2}-LHA neurons from sham controls and SNI mice. (**C** and **D**) The sample cumulative fraction (left) and bar plots (right) indicate the frequency (**C**) and the amplitude (**D**) of mEPSC in sham and SNI mice (n = 16 and 15 neurons from 5 mice per group). Two-tailed *t*-test; t_{29} = 3.269, ***p* = 0.0028 (**C**); t_{29} = 4.048, ****p* = 0.0004 (**D**). (**E**) Sample traces of mIPSC in mNAcSh^{D2}-LHA neurons from sham controls and SNI mice. (**F** and **G**) The representative cumulative fraction (left) and bar plots (right) show the frequency (**F**) and the amplitude (**G**) of mIPSC in sham and SNI mice (n = 16 and 15 neurons from 5 mice per group). Two-tailed *t*-test; t_{29} = 0.3159, *p* = 0.7543 (**F**); t_{29} = 1.693, *p* = 0.1013 (**G**). (**H**) Schematic diagram for projection-specific labeling of mNAcSh^{D2}-VP neurons (top) and the experimental time course (bottom). (**I**) Representative traces of mEPSC in mNAcSh^{D2}-VP neurons from sham controls and SNI mice. (**J** and **K**) The cumulative fraction (left) and bar plots (right) indicate the frequency (**J**) and the amplitude (**K**) of mEPSC in sham and SNI mice (n = 18 neurons from 5 mice per group). Two-tailed *t*-test; t_{34} = 0.6367, *p* = 0.5286 (**J**); t_{34} = 0.7687, *p* = 0.4474 (**K**). (**L**) Sample traces of mIPSC in mNAcSh^{D2}-VP neurons from sham and SNI mice. (**M** and N) The representative cumulative fraction (left) and bar plots (right) show the frequency (**M**) and the amplitude (**N**) of mIPSC in sham and SNI mice (n = 18 neurons from 5 mice per group). Two-tailed *t*-test; t_{34} = 3.482, ***p* = 0.0014 (**M**); t_{34} = 1.986, *p* = 0.0552 (**N**). Data are represented as mean ± SEM.

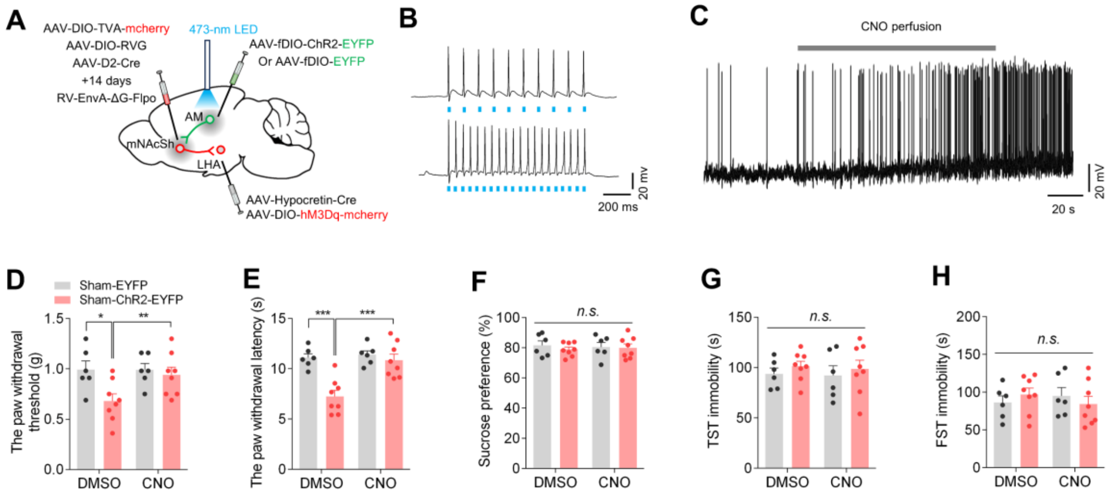

Supplemental Figure 8. Chemogenetic activation of LH orexinergic neurons reversed the pain hypersensitivity induced by optogenetic activation of AM-mNAcSh^{D2}-LHA neurons. (**A**) Schematic diagrams of chemogenetic activation of LH orexinergic neurons and optogenetic activation of the AM-mNAcSh^{D2}-LHA neurons. (**B**) Representative recordings of action potential firing of ChR2-expressing AM-mNAcSh^{D2}-LHA neurons in response to 10 Hz (top) and 20 Hz (bottom) light photostimulation. (**C**) Whole-cell current-clamp recording from hM3Dq-expressing LHA orexinergic neurons. A brief bath application of 10 μM CNO (gray line) increased the action potential firing. (**D** and **E**) Mechanical paw withdrawal threshold (**D**) and thermal paw withdrawal latency (**E**) in sham-operated mice received blue light stimulation with (CNO) or without (DMSO) chemogenetic activation of LH orexinergic neurons (n = 6-8 mice per group). Two-way repeated-measures ANOVA with Bonferroni's multiple comparisons test, interaction $F_{(1, 12)}$ = 6.052, p = 0.0300 (**D**); interaction $F_{(1, 12)}$ = 11.34, p = 0.0056 (**E**); *p < 0.05, **p < 0.01, ***p < 0.001. (**F-H**) Depressive-like behaviors in different groups. Percentage of sucrose water consumption in SPT (**F**), immobility time in TST (**G**), and immobility time in FST (**H**) (n = 6-8 mice per group). Two-way repeated-measures ANOVA with Bonferroni's multiple comparisons test, interaction $F_{(1, 12)}$ = 0.1605, p = 0.6958 (**F**); interaction $F_{(1, 12)}$ = 0.003829, p = 0.9517 (**G**); interaction $F_{(1, 12)}$ = 3.555, p = 0.0838 (**H**). Data are represented as mean ± SEM.

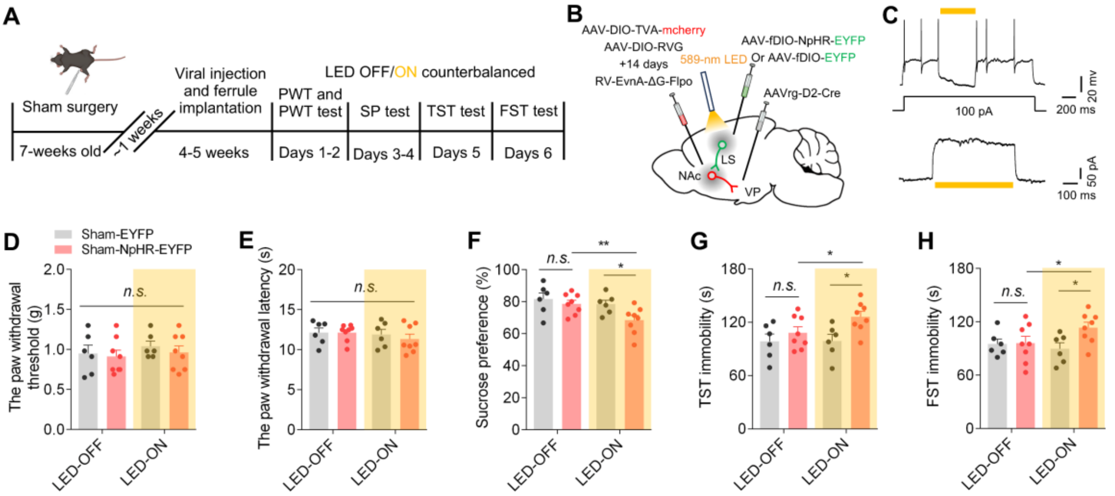

Supplemental Figure 9. Selective inhibition of the LS-mNAcSh^{D2}-VP pathway induced depressive-like behaviors in sham mice. (**A** and **B**) Schematic showing experimental timelines (**A**) and viral injection (**B**). (**C**) Whole-cell recording showed that yellow light inhibited action potentials (top) and induced outward current (bottom) in NpHR-expressing neurons. (**D**, E) Mechanical paw withdrawal threshold (**D**) and thermal paw withdrawal latency (**E**) in sham mice with optogenetic inhibition of LS-mNAcSh^{D2}-VP neurons (n = 6-8 mice per group). Two-way repeated-measures ANOVA with Bonferroni's multiple comparisons test; interaction $F_{(1, 12)} = 0.0856$, $p = 0.7748$ (**D**); $F_{(1, 12)} = 0.2705$, $p = 0.6125$ (**E**). (**F**-**H**) Percentage of sucrose water consumption in SPT (**F**), immobility time in TST (**G**), and immobility time in FST (**H**) in different groups of mice (n = 6-8 mice per group). Two-way repeated-measures ANOVA with Bonferroni's multiple comparisons test; time factor $F_{(1, 12)} = 8.879$, $p = 0.0115$ (**F**); $F_{(1, 12)} = 4.750$, $p = 0.0499$ (**G**); interaction $F_{(1, 12)} = 4.845$, $p = 0.0480$ (**H**); *$p < 0.05$, **$p < 0.01$. Data are represented as mean ± SEM.

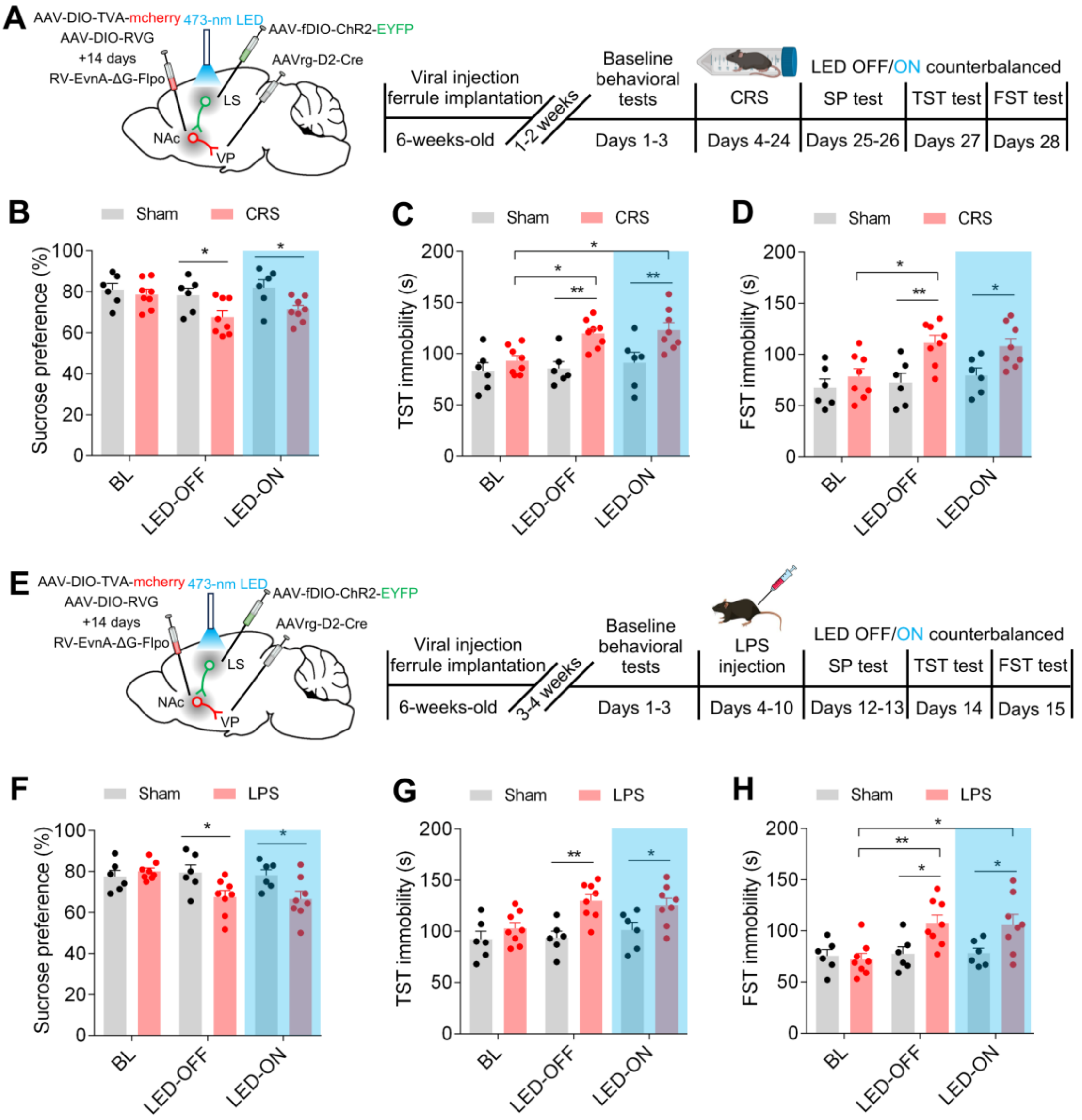

Supplemental Figure 10. Optogenetic activation of LS-mNAcSh^{D2}-VP neurons could not change the depressive-like behaviors resulting from either CRS or LPS injection. (**A**) Schematic showing viral injection (left) and experimental timelines (right) for testing the effects of optogenetic activating LS-mNAcSh^{D2}-VP neurons on depressive-like behaviors in CRS mice. (**B**-**D**) Percentage of sucrose water consumption in SPT (**B**), immobility time in TST (**C**), and immobility time in FST (**D**) in sham mice and CRS mice before and during LED illumination (n = 6-8 mice per group). Two-way repeated-measures ANOVA with Bonferroni's multiple comparisons test; group factor $F_{(1, 12)}$ = 13.84, p = 0.0029 (**B**); $F_{(1, 12)}$ = 19.99, p = 0.0008 (**C**); $F_{(1, 12)}$ = 19.11, p = 0.0009 (**D**); *p < 0.05, **p < 0.01. (**E**) Schematic showing viral injection (left) and experimental timelines (right). (**F**-**H**) The effects of optogenetic activating LS-mNAcSh^{D2}-VP neurons on the percentage of sucrose water consumption in SPT (**F**), immobility time in TST (**G**), and immobility time in FST (**H**) in sham mice and LPS-treated mice (n = 6-8 mice per group). Two-way repeated-measures ANOVA with Bonferroni's multiple comparisons test; interaction $F_{(2, 24)}$ = 3.729, p = 0.0389 (**F**); group factor $F_{(1, 12)}$ = 20.21, p = 0.0007 (**G**); group factor $F_{(1, 12)}$ = 8.329, p = 0.0137 (**H**); *p < 0.05, **p < 0.01. Data are represented as mean ± SEM.

Supplemental References

1. Gao C, et al. Two genetically, anatomically and functionally distinct cell types segregate across anteroposterior axis of paraventricular thalamus. *Nat Neurosci*. 2020;23(2):217-228.
2. Deng J, et al. The Parabrachial Nucleus Directly Channels Spinal Nociceptive Signals to the Intralaminar Thalamic Nuclei, but Not the Amygdala. *Neuron*. 2020;107(5):909-923.
3. Fan Z, et al. Neural mechanism underlying depressive-like state associated with social status loss. *Cell*. 2023;186(3):560-576.
4. Lu J, et al. An entorhinal-visual cortical circuit regulates depression-like behaviors. *Mol Psychiatry*. 2022;27(9):3807-3820.
5. Liu D, et al. An Ascending Excitatory Circuit from the Dorsal Raphe for Sensory Modulation of Pain. *J Neurosci*. 2024;44(4): e0869232023.
6. Tang QQ, et al. Direct paraventricular thalamus-basolateral amygdala circuit modulates neuropathic pain and emotional anxiety. *Neuropsychopharmacology*. 2024;49(2):455-466.
7. Marcinkiewcz CA, et al. Serotonin engages an anxiety and fear-promoting circuit in the extended amygdala. *Nature*. 2016;537(7618):97-101.
8. Zhang Y, et al. Targeting thalamic circuits rescues motor and mood deficits in PD mice. *Nature*. 2022;607(7918):321-329.
9. Huang J, et al. A neuronal circuit for activating descending modulation of neuropathic pain. *Nat Neurosci*. 2019;22(10):1659-1668.

10. Gu X, et al. Neurons in the caudal ventrolateral medulla mediate descending pain control. *Nat Neurosci*. 2023;26(4):594-605.

11. Xu ZZ, et al. Resolvins RvE1 and RvD1 attenuate inflammatory pain via central and peripheral actions. *Nat Med*. 2010;16(5):592-597.

12. Yang Y, et al. Ketamine blocks bursting in the lateral habenula to rapidly relieve depression. *Nature*. 2018;554(7692):317-322.

13. Ohgi Y, et al. Effects of antidepressants on alternations in serum cytokines and depressive-like behavior in mice after lipopolysaccharide administration. *Pharmacol Biochem Behav*. 2013;103(4):853-859.

14. Han Q, et al. SHANK3 Deficiency Impairs Heat Hyperalgesia and TRPV1 Signaling in Primary Sensory Neurons. *Neuron*. 2016;92(6):1279-1293.

15. Hargreaves K, et al. A new and sensitive method for measuring thermal nociception in cutaneous hyperalgesia. *Pain*. 1988;32(1):77-88.

16. Yang H, et al. Pain modulates dopamine neurons via a spinal-parabrachial-mesencephalic circuit. *Nat Neurosci*. 2021;24(10):1402-1413.
